# Biglycan regulates bone development and regeneration

**DOI:** 10.3389/fphys.2023.1119368

**Published:** 2023-02-16

**Authors:** Reut Shainer, Vardit Kram, Tina M. Kilts, Li Li, Andrew D. Doyle, Inbal Shainer, Daniel Martin, Carl G. Simon, Jinyang Zeng-Brouwers, Liliana Schaefer, Marian F. Young, Genomics and Computational Biology Core

**Affiliations:** ^1^ Molecular Biology of Bones and Teeth Section, National Institute of Dental and Craniofacial Research, National Institutes of Health, Bethesda, MD, United States; ^2^ NIDCR Imaging Core, National Institute of Dental and Craniofacial Research, National Institutes of Health, Bethesda, MD, United States; ^3^ Department Genes-Circuits-Behavior, Max Planck Institute for Biological Intelligence, Martinsried, Germany; ^4^ NIDCD/NIDCR Genomics and Computational Biology Core, National Institutes of Health, Bethesda, MD, United States; ^5^ Biosystems and Biomaterials Division, National Institute of Standards and Technology, Gaithersburg, MD, United States; ^6^ Pharmazentrum Frankfurt, Institut für Allgemeine Pharmakologie und Toxikologie, Klinikum der Goethe-Universität Frankfurt am Main, Frankfurt, Germany

**Keywords:** periosteum, biglycan, extracellular matrix, fracture, bone, cartilage

## Abstract

Endochondral bone development and regeneration relies on activation and proliferation of periosteum derived-cells (PDCs). Biglycan (Bgn), a small proteoglycan found in extracellular matrix, is known to be expressed in bone and cartilage, however little is known about its influence during bone development. Here we link biglycan with osteoblast maturation starting during embryonic development that later affects bone integrity and strength. Biglycan gene deletion reduced the inflammatory response after fracture, leading to impaired periosteal expansion and callus formation. Using a novel 3D scaffold with PDCs, we found that biglycan could be important for the cartilage phase preceding bone formation. The absence of biglycan led to accelerated bone development with high levels of osteopontin, which appeared to be detrimental to the structural integrity of the bone. Collectively, our study identifies biglycan as an influencing factor in PDCs activation during bone development and bone regeneration after fracture.

## Introduction

Skeletal development proceeds through two different ossification processes. Craniofacial bones develop through intramembranous ossification by direct differentiation of the mesenchymal stem/progenitor cells into osteoblasts. Development of the long bones on the other hand occurs *via* endochondral ossification, in which the mesenchymal stem/progenitor cells differentiate into chondrocytes and lay down a cartilage template, which is later replaced by osteoblasts that are responsible for bone formation (Berendsen and Olsen, 2015; Salhotra et al., 2020).

The periosteum is a thin highly vascularized membrane surrounding the bone that serves as an attachment site for tendons, ligaments and muscles. The periosteum is composed of an external fibrous layer containing fibroblasts and an inner cambium layer containing progenitor cells, known as periosteum derived-cells (PDCs), that allow bone growth and remodeling (Marsell and Einhorn, 2011; Colnot et al., 2012; Roberts et al., 2015).

Bone fracture healing involves a complex sequence of physiological events, which is similar to embryonic bone development except that it has an inflammation phase after the fracture that is necessary for the regeneration progress. In general, fracture healing through endochondral bone formation involves 4 phases: 1) inflammation, 2) proliferation, 3) callus formation and 4) bone remodeling. The initial fracture leads to a hematoma followed by an inflammatory response. Inflammatory cells migrate to the fracture area and secrete stimulatory factors including IL-1, IL-6, and bone morphogenetic proteins (BMPs) to promote angiogenesis and to recruit skeletal progenitor cells from the periosteum. After the inflammation phase, a callus forms due to a massive proliferation of PDCs leading to cellular condensation and chondrogenic differentiation. Recent evidence show that the periosteum is the major source of progenitor cells needed for the bone repair process (Marecic et al., 2015; Debnath et al., 2018; Duchamp de Lageneste et al., 2018). In the next phase of bone healing, the chondrocytes in the callus become hypertrophic and blood vessels and osteoblasts from periosteal regions enter to replace the cartilage template and form the woven bone. In the final phase of the fracture healing, the woven bone remodels through osteoclast-osteoblast coupling to create the mature lamellar bone.

The extracellular matrix (ECM) of bone plays a pivotal role in tissue integrity and strength. The organic ECM is comprised mainly of collagen type I (90%), which is arranged in fibrils that become mineralized to provide a scaffold for bone cells and ultimately determines the bone strength and integrity. Other components of the ECM are the non-collagenous proteins (10%) which mainly include γ-carboxyglutamic-containing proteins, small integrin-binding ligand N-linked glycoproteins (SIBLINGs) and proteoglycans, including small leucine-rich proteoglycans (SLRPs), that also contribute to bone structure and strength (Lin et al., 2020).

Biglycan (Bgn) is a member of the SLRP family and is highly abundant in the ECM of a variety of tissues, including bone, cartilage and tendon (Fisher et al., 1983; Bianco et al., 1990). During skeletal development, high levels of Bgn mRNA are detected in areas of endochondral and membranous bone formation (Kram et al., 2020). To study the role of Bgn in the skeleton, a knockout (KO) mouse, globally deficient in *Bgn*, was generated. Although *Bgn*-deficient mice appear normal at birth, they display a phenotype characterized by reduced bone mass and age-dependent osteopenia (Xu et al., 1998; Chen et al., 2002). In this study, we explored the role of Bgn from early stages of bone development and during fracture healing. Lack of Bgn influenced embryonic osteoblast differentiation, which later resulted in structural changes in the bone, including reduced integrity and strength. In addition, the fracture healing cascade in *Bgn* KO mice was compromised in multiple ways. Unlike wild-type (WT) mice, *Bgn-*deficient mice showed a reduced response to injury during the inflammatory phase, which led to decreased periosteal expansion, resulting in a smaller callus around the fracture site that then mineralize and remodel faster, generating new bone that was structurally abnormal. Analyzing the expression pattern of Bgn showed that it was dramatically upregulated in the periosteum in response to fracture. Using a novel 3D system, we found that under conditions that stimulate osteogenic differentiation, WT PDCs underwent endochondral ossification by forming cartilage structures *in vitro* followed by mineralization *in vivo*. In the 3D scaffolds containing *Bgn* KO PDCs, cartilage differentiation was reduced which resulted in abnormal accelerated mineralization. Overall, our results demonstrate that *Bgn* deletion impairs bone development and regeneration and may do so by regulating the cartilage phase preceding bone formation.

## Materials and methods

### Mice

C57BL/6J and C57BL/6-Tg (CAG-EGFP)1Osb/J male mice were obtained from The Jackson Laboratory. *Bgn*-deficient (KO) mice were generated as previously described (Xu et al., 1998) all mice were bred and housed at the NIDCR/NIH/DHHS animal facility with standard conditions and genotyped in our laboratory. All procedures using mice were approved by the NIDCR/NIH/DHHS ACUC (protocol numbers 18-865, 18-871).

### Primary culture of PDCs

After euthanasia, femurs and tibias were dissected from 6 to 7 week-old WT or *Bgn* KO male mice. Muscle and connective tissue were removed under sterile conditions and the epiphyses were coated with 5% low melting point agarose (Invitrogen) to protect the cartilage from digestion. PDCs were isolated by 1 h digestion in 3 mg/mL collagenase type II (Gibco) and 4 mg/mL dispase (Gibco) in α-minimal essential medium (αMEM) with 3% glutaMAX (Gibco) at 37°C (van Gastel et al., 2012; van Gastel et al., 2014). The cell suspension was passed through a 70 μm cell strainer (Falcon) and plated in 25 cm^2^ flasks with growth medium composed of αMEM (Gibco) supplemented with 20% FBS (Gemini Bio Products), 100 U/mL each of penicillin and streptomycin and 2 mM glutaMAX (Gibco), and the medium was replenished twice a week until cells reached confluence.

### Preparation of 3D culture system

When PDC cultures reached 80%–90% confluency (10–14 days post isolation), the cells were trypsinized and seeded in RAFT™ 3D 96 well cell culture plates according to the manufacturer’s instructions (RAFT™ 3D Cell Culture bundle Kit, Lonza) at a concentration of 120,000 cells/well. Briefly, cells were seeded in a chilled mixture of collagen solution containing x10 MEM medium, 2 mg/mL rat tail collagen type I and neutralizing solution and incubated for 18 min at 37°C to form a hydrogel. Next, an absorber device was placed on top of the hydrogel for 15 min to condense and concentrate the mix of cells and collagen to create the RAFT™ disc. 0.24 mL/well growth medium was immediately added and thereafter replaced twice a week. To induce osteogenic differentiation, 10^–8^ M dexamethasone, 100 μM L-ascorbic acid phosphate and 2 mM β-glycerophosphate were added to the growth medium containing only 10% FBS for 14 days.

### Fractures and 3D scaffold transplantation

For all surgeries, 6–7 week-old WT or *Bgn* KO male mice were anesthetized with 2%–3% isoflurane. 1 mg/kg of buprenorphine SR-LAB (ZooPharm) was administrated subcutaneously before the procedure and 72 h post-surgery. For open stabilized fractures, the right hindleg was shaved and scrubbed with 10% povidone-iodine and alcohol solutions. A 5 mm medial parapatellar incision was created and the patella was dislocated to expose the femoral condyle. A hole was burred into the femoral epiphysis using a 25 gauge (25 G) needle and enlarged using a 23 G needle. A 26 G cannula (Millpledge Veterinary) was inserted into the femoral shaft through the burred hole. The muscles and tendons were shifted to expose the bone shaft and a horizontal fracture was created at the center of the shaft using surgical scissors. The soft tissue was repositioned and the protruding end of the needle was cut off. The incision was closed using 5-0 absorbable sutures (Ethicon). X-ray was performed to visualize the fracture (Faxitron® Ultra Focus). For subcutaneous transplantation, a small incision was made in the skin on the back of the mouse. 3D scaffolds with un-differentiated PDCs (that served as control) or differentiated PDCs were implanted subcutaneously and the incision was closed using 5-0 absorbable sutures (Ethicon). The 3D scaffolds were analyzed at time points 0 (the day of implantation), and 4 and 8 weeks after implantation.

### Micro-computed tomography (µCT)

The 3D scaffolds recovered after implantation and mice femurs before and after fracture were fixed in Z-fix (170; Anatech, LTD) for 24 h at room temperature (RT) and stored in 70% ethanol. 3D transplants were scanned at 45 kV, 200 μA, 600 ms integration time (IT), 14.8 µm voxel size. Femurs were scanned at 70 kV, 85 μA, 300 ms IT, 10 µm resolution (µCT50; Scanco Medical AG, Brüttisellen, Switzerland). Mineralized tissues were reconstructed using the global approach and segmented by a global thresholding software (Scanco Medical AG, Brüttisellen, Switzerland). Standardized nomenclature was used for the bone parameters measured. For the femurs, trabecular parameters were measured at the secondary spongiosa of the distal metaphysis and cortical parameters were determined in a 1 mm ring at the mid-diaphyseal region according to previously published guidelines (Bouxsein et al., 2010). The callus of the fractured femurs was analyzed along the entire shaft of the bone. Cross section measurements of the callus were calculated based on the entire stack of 2D images.

### Histology and imaging

For scanning electron microscopy (SEM), 3D scaffolds were fixed at 4°C overnight with Karnovsky fixative (Electron Microscopy Sciences) followed by three washes with phosphate buffered saline (PBS) and dehydrated in a series of ethanol washes (0, 30, 50, 70, 85, 95, and 100%), with each wash conducted 3 times for a total of 15 min. The samples were then critical point dried (Autosamdri-814, Tousimis), mounted and sputter-coated with gold (75 mA for 60 s, Denton Vacuum Desk II) prior to imaging (2 kV, 10 Ua, S-4700-II FE-SEM, Hitachi). For histology, immunofluorescent and second harmonic generation (SHG) imaging, 3D scaffolds or femurs were decalcified prior to paraffin-embedding.

Femur sections were deparaffinized and rehydrated prior to SHG imaging of type I collagen. SHG imaging was performed on a Nikon A1R MP + HD confocal system using a ×40 Apo LWD objective (N.A. 1.15) in resonant scanning mode (512 × 512, 4X line average scanning). The two-photon beam excitation was provided by a Chameleon Vision II laser tuned to 820 nm (Coherent). The non-descanned detector used a 400–480 band pass emission filter. Z-stacks were taken of the middle 10 or 15 microns of 20 or 30 micron-thick tissue slices, respectively. Image tiles were created in a ∼250–500 µm wide by 1800 µm long region centered around the femur center. Femur sections were oriented to match the two-photon beams polarity (parallel with the *Y*-axis). After acquisition, images were denoised using NIS Elements Denoise A.I. software (Nixon). Line scans depicted in figures show SHG signal intensity normalized to the brightest pixels for comparison on a similar scale.

To analyze SHG fibril differences between WT and KO femurs, ctFIRE and curve align standalone plugins for MatLab (Bredfeldt et al., 2014) were used with the following settings: Min fiber length 100 pixels, Max fiber width 15.

For Bgn immunochemistry, deparaffinized and rehydrated sections were incubated for 1 h at 37°C with ABCase (Seikagaku biobusiness corp.), followed by antigen retrieval (Unitrieve, Innovex), and quenching of endogenous peroxidase activity with dual endogenous enzyme block (Dako). The sections were blocked with 10% normal goat serum in PBS for 1 h at 37°C and incubated with Bgn rabbit antisera (from Dr. Larry W. Fisher, NIH, ref. LF-159) 1:600 diluted in the blocking buffer overnight at 4°C. The sections were washed and incubated with Super PicTure™ Polymer detection kit (Invitrogen) for 20 min at RT and detected with ImmPACT™ AEC (Vector laboratories). Slides were scanned using an Aperio ScanScope slide scanner.

For F4/80, Ym1, and iNOS immunohistochemistry, deparaffinized and rehydrated sections were incubated for 48 h at 55°C with citrate buffer, pH 6.0 (BIOZOL, Germany). The sections were blocked with 3% H_2_O_2_ in PBS for 10 min and with Protein Block Serum-free solution (Dako, X0909) for 20 min. This was followed by incubation with rat anti-mouse F4/80 antibody (1:250, Biorad, MCA497G), rabbit anti-Ym1 antibody (1:100, Abcam, ab93034) or rabbit anti-iNOS (1:500, Enzo Life Sciences, ADI-905-431–1) diluted in antibody diluent (Dako S3022) overnight at 4°C. The sections were washed and incubated with Histofine Simple Stain Mouse MAX PO (Rat) (Nichirei Biosciences INC, 414311F) for F4/80, or with Histofine Simple Stain Mouse MAX PO (Rabbit) (Nichirei Biosciences INC, 414341F) for Ym1 and iNOS, for 30 min at RT and detected with Vector DAB Substrate (Vector laboratories, SK4100). Mayer’s hematoxylin was used as counterstain. The protein expression levels were determined by the reciprocal intensity of the chromogen stain using the open source ImageJ Fiji software (http://fiji.sc/Fiji).

For aggrecan (ACAN) and osteopontin (OPN) immunofluorescence, deparaffinized and rehydrated sections were incubated for 1 h at 37°C with ABCase (Seikagaku biobusiness corp). Following antigen retrieval (Unitrieve, Innovex), and incubation with 0.1% sodium borohydride, the sections were blocked with 10% normal goat serum in PBS for 1 h at 37°C, and incubated 1:200 with either rabbit anti-ACAN antibody (Millipore, ref. AB1031) or rabbit anti-OPN antibody (IBL, ref. 18,621) overnight at 4°C. The sections were washed and incubated with secondary antibody, anti-Rabbit IgG conjugated to Rhodamine Red 1:500 (Jackson, ref. AB_2340614) and DAPI 1:500 (Thermo Fisher Scientific, ref. D21490) for 1 h at RT. Finally, the slides were washed, mounted and imaged by A1R-MP + HD multiphoton confocal system (Nikon). Tiled (2 × 2, 6 × 6 or 7 × 7) Z-stacks (every 1.0 µm) were taken using a ×40 Plan Fluor (N.A 1.3) oil immersion objective in resonant scanning mode (×512512, 4X line average scanning) using the 405 nm and 561 nm lasers. Images were denoised using NIS Elements Denoise A.I. software after acquisition (Nixon). For quantification, we applied a Li threshold and then captured the mean fluorescence intensity of included area of the image using ImageJ Fiji software.

For osteoclast analysis, deparaffinized sections of fractured bones were stained with TRAP (Sigma).

Staining with hematoxylin and eosin (H&E) was performed using standard protocols. For periosteal width analysis, H&E stained slides were scanned using an Aperio ScanScope slide scanner and the entire enlarged periosteum was outlined, measured and the expanded area analyzed by Image Pro 7.0 software (Media Cybernetics Inc., United States). This resulted in five data points for each genotype used.

### RNA isolation, qRT-PCR and RNAseq

Femoral shafts were isolated and immediately frozen in liquid nitrogen. The bones were centered in a tissue tube (Covaris) under liquid nitrogen and crushed using a CP02 cryoPREP Dry Pulverizer. Total mRNA was extracted and purified from the pulverized tissue using TriPure (Sigma, United States) followed by RNeasy mini kit (Qiagen) hybrid protocol.

Total mRNA from cultured cells was extracted and purified using RNeasy mini kit (Qiagen).

Total mRNA was converted to cDNA using iScript cDNA Synthesis Kit (Bio-Rad) and qRT-PCR analysis was performed using iQ SYBR Green Supermix (Bio-Rad). Target gene (Bgn: F: 5′-AGA​CAA​ACC​GAC​AGC​CTG​ACA​AC-3′, R: 5′-GCC​AGC​AGC​AAG​GTG​AGT​AGC-3′) was normalized to S29 (F: 5′-GGA​GTC​ACC​CAC​GGA​GTT​CG-3′, R: 5′-GGA​AGC​AGC​TGG​CGG​CAC​ATG-3′) and relative expression data was calculated using the ΔΔCt method.

For RNA seq procedures, RNA was transcribed by Superscript IV (Thermo Fisher Scientific) and full-length 2nd strand cDNA amplified by LongAmp DNA polymerase (New England BioLabs). Sequencing libraries were prepared using a Nextera XT kit (Illumina), individually barcoded, pooled to a 2 nM final concentration, and sequenced on a NextSeq500 or NextSeq2000 instrument (Illumina) using 37 × 37 paired-end (NextSeq 500) or 55 × 55 (NextSeq 2000) paired-end read configurations. After sequencing, the base-called demultiplexed (fastq) reads from multiple sequencing runs were merged when appropriate and read qualities were determined using FastQC (v0.11.2), aligned to the GENCODE M11 mouse genome (GRCm38.p4) and gene counts generated using STAR (v2.5.2a). Post-alignment qualities were generated with Picard Tools RnaSeqMetrics (v1.129). An expression matrix of raw gene counts was generated using R and filtered to remove low count genes (defined as those with less than five reads in at least one sample). The filtered expression matrix was used to generate a list of differentially expressed genes (DEGs) between the sample groups using three statistical methods: DESeq2, EdgeR, and Limma-voom.

### Single cell RNA seq

For single-cell RNA sequencing (scRNA seq), WT and *Bgn* KO embryos (E16.5) were used. After euthanasia, the posterior limbs were dissected and connective tissues were removed under sterile conditions. The bones were dissected into small pieces and digested with 2 mg/mL collagenase IV (Gibco) in Advanced DMEM (supplement with 1% Glutamine) for 40 min at 37°C with manual shaking every 10 min. When the dissociation procedure was complete, the cell-suspension was centrifuged, supernatants were carefully removed and the cell pellet suspended in PBS containing 0.1% BSA. The cells were then filtered using Flowmi filter tips (Bel-Art Products, #H13680-0040) to remove possible undissociated cells and debris. The cells were counted and resuspended to a final concentration of 1 × 10^6^ cells/mL.

The single-cell suspension was loaded onto the droplet-based single-cell barcoding system (10x Chromium Controller, 10x Genomics) and a Chromium NextGEM Single Cell 3’ Reagent Kit v3.1 (10x Genomics) was used to prepare single-cell, barcoded 3’ cDNA libraries according to the manufacturer’’s instructions. The libraries were sequenced on a NextSeq2000 instrument (Illumina). The sequenced data was processed by CellRanger (v5.0.0, filtering, barcode and UMI counting) using default command line options and gene-barcode matrices were generated. The sequenced reads were aligned to the mouse genome assembly provided by 10X Genomics (10X Genomics reference mm10-2020-A), based on Ensembl annotation. Downstream data analysis was performed on the CellRanger cell-gene filtered matrix, using the Seurat R package V4 (Hao et al., 2021). Seurat objects were generated for each of the four samples (2 WT, two *Bgn* KO), which were then merged into a single Seurat object. Cells with unusual numbers of genes (<200), UMI count (>15,000) or percentage of mitochondrial genes (>20%) were filtered out. The data was then normalized by the “LogNormalization” method implemented in the Seurat package (scale factor = 10,000) and scaled using Seurat’s default settings. The 2,000 top variable genes were identified using the “vst” method implemented in Seurat. Linear dimensional reduction (PCA) was performed, followed by batch correction using “Harmony” (“RunHarmony” command, using default settings) (Korsunsky et al., 2019). Nearest Neighbor analysis and clustering were performed using the Harmony embeddings. The clusters were visualized using Uniform Manifold Approximation and Projection (UMAP) (Becht et al., 2018). The DEGs between the clusters were identified using the Wilcoxon Rank Sum test implemented in Seurat’s “FindAllMarkers” command. The cell identities were then assigned according to the markers identified (Supplementary Tables S1, S2). Cell types that were over clustered were merged (e.g., mast cells). For pseudotime trajectory analysis, a new Seurat object containing the osteoblasts clusters (1–4) and the skeletal progenitor cell was generated. The single cell trajectory and branching point were detected using the reversed graph embedding algorithm implemented in Monocle3 R package (Qiu et al., 2017) using default parameters. The root of the trajectory was defined as the list of the skeletal progenitor cells. The full code for the analysis and figures will be found in https://github.com/ishainer/R_shainer_et_al_2022/. The raw scRNA seq data is available through the NCBI GEO repository, accession number GSE192542, upon publication (token will be given upon reviewer’s request).

### Serum cytokines evaluation

Mouse serum was analyzed from peripheral blood, obtained by retro-orbital bleeding, 24 h post fracture. Circulating cytokine levels were determined by flow cytometry using a mouse inflammation CBA kit (BD Bioscience, BD 552364) according to the manufacturer’s protocol. Briefly, a mix of six bead populations coated with antibodies specific for IL-6, IL-10, MCP-1, IFN-γ, TNF, and IL-12p70 were incubated for 2 h at RT with serum samples and with PE conjugated detection antibodies to form sandwich complexes. PE fluorescence intensity for each of the antibodies was measured by Flow Cytometer and FCAP Array™ software was used to generate the results.

### Micro indentation

Femurs were embedded without demineralization in SamplKwick fast cure acrylic compound (Burhler). 7 mm thick cross-sections were cut from the bone’s mid-shaft using a diamond saw. The samples were ground and polished with Micro-Mesh sanding sheets followed by 0.25 µm diamond paste. Indents were preformed using HMV-G21DT micro-hardness testing machine (Shimadzu) with a diamond Vickers microindenter tip using 0.05 N of applied force for 10 s before unloading. 3-4 indents were performed for each sample and the results were averaged.

### Statistical analysis

Differences were examined by two-tailed Student’s t-test for comparing two groups and by either one-way or two-way analysis of variance (ANOVA) test for comparing multiple groups. When significant differences were indicated by ANOVA, group means were compared to establish the source of the differences. *p* < 0.05 was considered statistically significant.

## Results

### Single-cell sequencing of mouse embryonic bones shows Bgn is important for osteoblast differentiation

**FIGURE 1:**
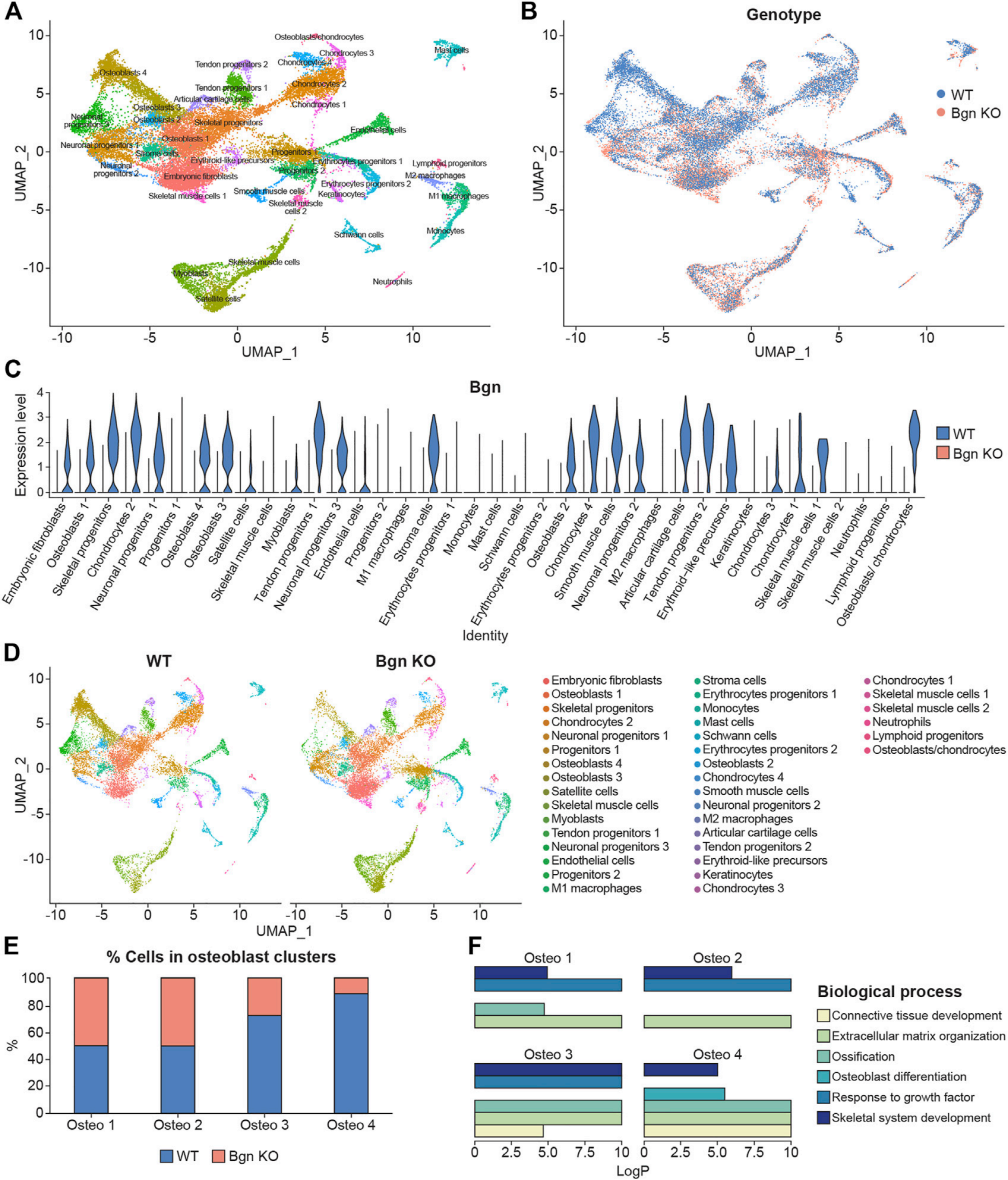
Single-cell sequencing of mouse embryonic bones shows Bgn is important for osteoblast differentiation. **(A)** UMAP visualization of transcriptional clusters derived from WT and *Bgn* KO cells (E16.5). Each point represents a single cell, colored according to cell type. The cells were clustered into 38 distinct types, which were defined according to their unique transcriptomes. See also Supplementary Table S1. **(B)** UMAP visualization of the cells, colored according to their genotype origin. See also Supplementary Figure S1A. **(C)** Violin-plot comparison of *Bgn* gene expression in the different clusters, split according to their genotype origin. See also Supplementary Figure S1B. **(D)** UMAP visualization of the 38 clusters, WT compared with *Bgn* KO cells. See also Supplementary Figure S2. **(E)** The percentage of WT and *Bgn* KO cells that populate the osteoblast clusters. **(F)** GO analysis of the genes in the different osteoblast clusters.

Bgn is an abundant matrix component expressed in skeletal tissues throughout bone development and maturation. To study the role of Bgn *in vivo*, a *Bgn*-deficient mouse line was generated (Xu et al., 1998). Although *Bgn* KO mice appear normal at birth, they display a phenotype characterized by reduced bone mass and age-dependent osteopenia. Lechner et al. (2006) measured the expression of *Bgn* mRNA in mouse hindlimbs during development and found that *Bgn* was present at E14, exhibited a striking 5-fold upregulation at E16, before dropping 5-fold by E18. Therefore, to understand when bone defects begin to arise in *Bgn* KO mice, we isolated cells from fetal hindlimbs of WT and *Bgn* KO embryos at 16.5 days of age, where *Bgn* transcript level is at its highest, and performed single-cell RNA sequencing (scRNA seq) analysis using the 10X Genomic’s Chromium platform. Clustering analysis of the sequenced cells revealed 38 transcriptionally distinct populations. The top 50 differentially expressed genes within each cluster were used to identify the cell-type represented by each cluster (Figure 1A; Supplementary Table S1). 14,781 WT cells and 15,054 *Bgn* KO cells were analyzed, and both genotypes expressed the same clustering pattern (Figure 1B; Supplementary Figures S1A, S2). *Bgn* was found to be expressed mainly in skeletal progenitor cells, osteoblasts, chondrocytes, stromal cells, embryonic fibroblasts, tendon cells and neuronal progenitor cell clusters (Figure 1C). *Bgn* mRNA wasn’t detected in *Bgn* KO cells as shown in Figure 1C and Supplementary Figure S1B. All the clusters included cells originating from both WT and *Bgn* KO animals, but the relative distribution of *Bgn* KO and WT cells differed in many of the clusters (Figure 1D; Supplementary Figure S2). From the total analyzed cells, 8.6% were chondrocytes. However, 5.6% came from WT cells and 3% came from *Bgn* KO cells. In addition, from the total analyzed cells, 18.6% were osteoblasts. In this cell type, 12.1% came from WT cells while only 6.5% came from *Bgn* KO cells. More specifically, in osteoblast clusters 1 and 2, the number of WT cells was almost equal to the number of *Bgn* KO cells but in the progression to osteoblast clusters 3 and 4 the number of *Bgn* KO cells dramatically decreased. In particular, osteoblast cluster 4 was comprised primarily of WT cells (88%) versus *Bgn* KO cells, which represented a minor fraction (12%) (Figures 1D–E; Supplementary Figure S2). Since the total number of cells that was analyzed equally represent both genotypes, the differences in specific cluster distribution most likely reflect the effect of *Bgn* deficiency on cell differentiation. To identify the differences between the 4 osteoblast clusters, enrichment analysis of biological pathways was performed on the differentially expressed genes. While the cells in all osteoblast clusters expressed genes that are involved in the ECM organization and the skeletal system development pathways, the genes specifically associated with the ossification pathway were highly expressed in osteoblast clusters 3 and 4, and genes associated with the osteoblast differentiation pathway were only found in osteoblast cluster 4 (Figure 1F). To further investigate the relationship between the osteoblast clusters, we analyzed their differentiation progress along a pseudotime trajectory using monocle3 (Qiu et al., 2017). The pseudotime trajectory was tested for the four osteoblast clusters, together with the skeletal progenitor cells, that were defined as the trajectory root. The global gene expression changes, enabled the detection of a single branching point leading the cell fate towards osteoblasts 3 and osteoblasts 4 (Supplementary Figures S1C, D), suggesting these clusters differentiate later than osteoblast cluster 2.

Taken together these data suggest that the differentiation and maturation levels of the osteoblasts are more advanced as they progress from osteoblast cluster 1 to osteoblast cluster 4. Cells from embryonic *Bgn* KO bone have less mature osteoblasts, less chondrocytes and more immature embryonic fibroblast cells (Figure 1D; Supplementary Figure S2), suggesting that Bgn is needed for early stages of bone development starting from the origin of the cells that generate the bone.

### Biglycan is needed for the structural integrity and the hardness of bone

To understand how the changes we found in the scRNA seq of embryonic bones could affect skeletal development, µCT was performed on mature 6 week-old mice.

**FIGURE 2:**
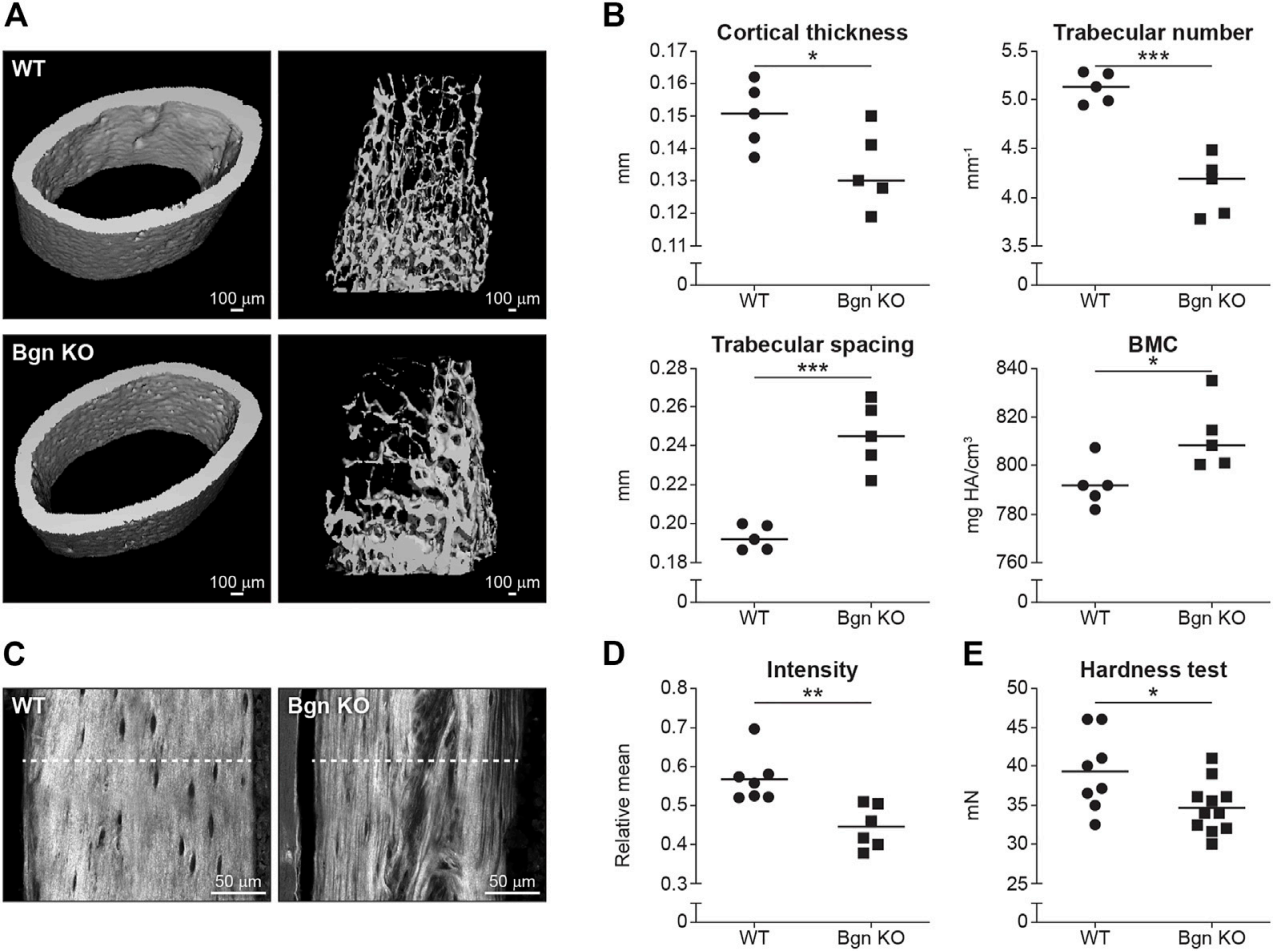
Bgn is needed for the structural integrity and the strength of bone. **(A)** 3D µCT reconstruction of femoral mid-diaphyseal cortical bone and the distal femoral metaphyseal bone from WT and *Bgn* KO mice (6 week-old), representative images. **(B)** Quantitative µCT analysis of cortical thickness, trabecular number, trabecular spacing, and Bone Mineral Content (BMC) (*n* = 5 per group). **(C)** Second harmonic generation (SHG) representative images of the collagen fibers in WT and *Bgn* KO femurs (6 week-old). **(D)** Quantitative SHG analysis of Type I collagen intensity. (*n* = 6/7 per group). **(E)** Micro indentation analysis of hardness of the femurs of WT and *Bgn* KO 6 week-old mice. (*n* = 8/11 per group).

Our data showed that there was a reduction in cortical thickness and trabecular number, and an increase in trabecular spacing and bone mineral content (BMC) in *Bgn* KO mice compared with WT mice (Figures 2A, B). Since 90% of the organic ECM of bone is collagen type I, second harmonic generation (SHG) microscopy was used to visualize and quantify the structural integrity of the collagen fibers in the *Bgn*-deficient bones. High collagen signature, as indicated by a consistently high even SHG intensity, with minor spacing between the collagen fibers was observed in the femurs of WT mice, whereas *Bgn* KO femurs showed significantly less of a collagen signature and more spacing between the fibers (Figures 2C, D). To understand how these molecular structural changes were affecting bone hardness, we assessed the strength of the bone by micro-indentation and found a reduction in the hardness of the *Bgn* KO bones compared with WT (Figure 2E). These results correlate with previous observations by Wallace et al. (2006) who used a 4-point bending test to demonstrate a reduction in the bone strength in Bgn KO bones compared to WT. Taken together, our findings show that in the absence of Bgn, compromised bone formation occurs that, ultimately, appears to affect collagen integrity, and subsequently biomechanical phenotype.

#### Lack of biglycan impairs bone healing

Considering bone defects were discovered in the mature bones of *Bgn* KO mice, we wanted to examine the skeletal remodeling process under the challenge of induced bone fracture. Bone healing involves a complex sequence of physiological events, starting with an inflammatory response at the fracture site, followed by activation and proliferation of skeletal stem cells in the periosteum and in the bone marrow. In the first phase of inflammation, inflammatory chemokines and cytokines are secreted to recruit inflammatory cells. Migration of macrophages into the fracture area has a major impact on the long term outcome of bone healing (Schlundt et al., 2018). Schaefer et al. (2005) previously showed in a sepsis model that Bgn, upon release from the ECM or from macrophages, acts as a proinflammatory factor and can boost inflammation by signaling through toll-like receptor (TLR) 2 and TLR4, which mediate the innate immunity.

**FIGURE 3:**
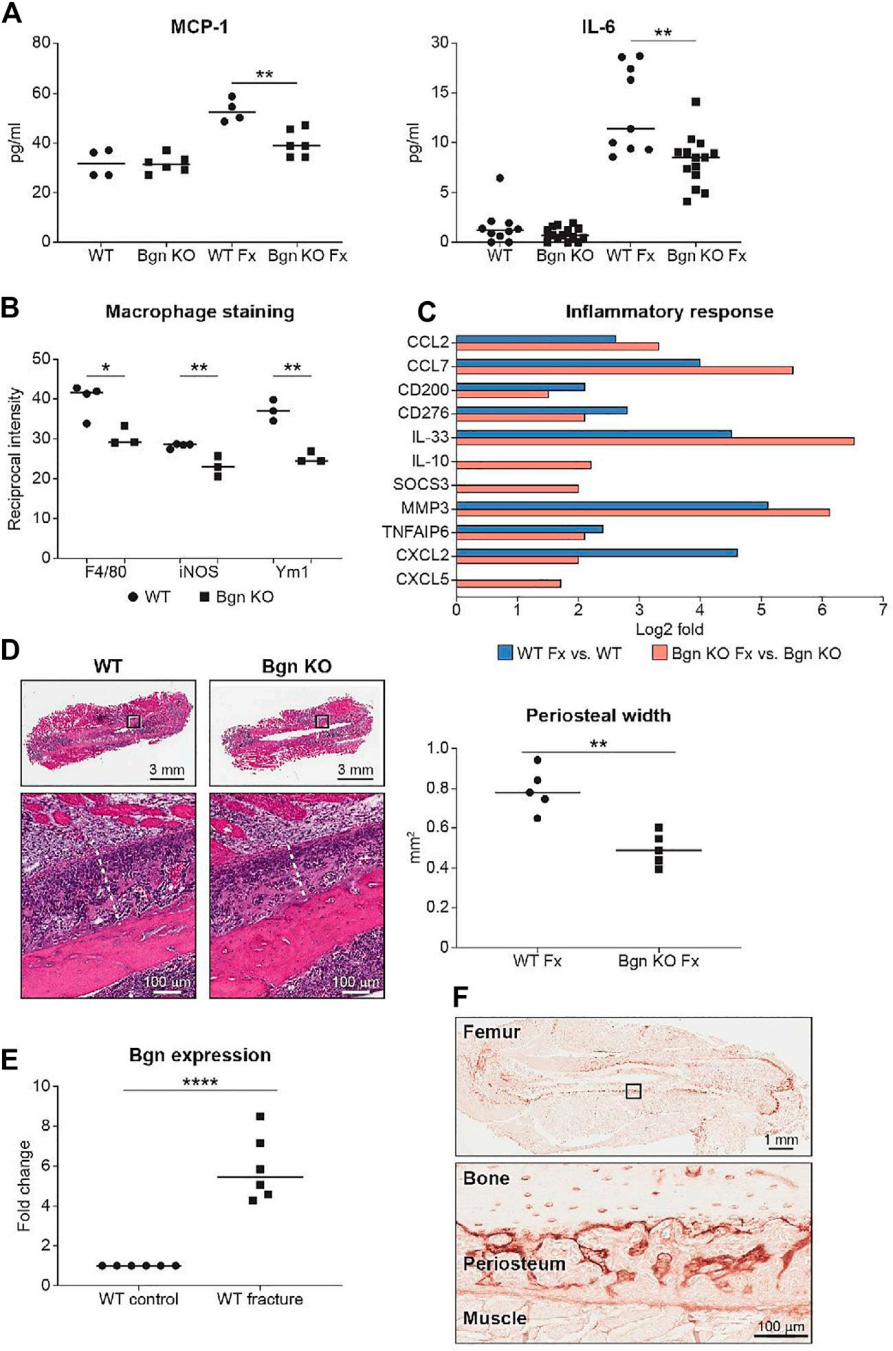
Bgn is needed for the inflammatory response after fracture. **(A)** Analysis of systemic cytokine secretion 24 h after fracture (Fx—Fracture). (For MCP-1, *n* = 4/6 per group; for IL-6, *n* = 10/14 per group) **(B)** Quantification of immunostaining for F4/80, iNOS and Ym1 around the fracture site 72 h after fracture (*n* = 3 per group). See also Supplementary Figure S3. **(C)** GO analysis of RNAseq data for genes involved in the inflammatory response before and 72 h after fracture (*n* = 6 per group). **(D)** Representative images and quantification of periosteal width 72 h after fracture (*n* = 5 per group). **(E)** Real-time PCR analysis of *Bgn* gene expression in WT bones before and 3 days after fracture (*n* = 6 per group). **(F)** Immunohistochemistry of Bgn in the periosteum 72 h after fracture, representative images (*n* = 5 per group).

To evaluate the onset of inflammation as a result of the injury, serum was obtained from peripheral blood 24 h after fracture. We analyzed the systemic inflammatory cytokine secretion and found a reduction in monocyte chemoattractant protein 1 (MCP-1) and IL-6 secretion in *Bgn* KO mice (Figure 3A). MCP-1 is a chemokine that regulates macrophage infiltration and is highly expressed in the periosteum in response to fracture. To understand how MCP-1 reduction impacts macrophage infiltration, fractured bones were collected 1–3 days after fracture and stained for different macrophage markers. Significant reduction in macrophage infiltration around the fracture site was found in *Bgn* KO compared with WT mice. Lower numbers of both F4/80^+^ and iNOS^+^ M1 macrophages were observed as early as 1 day after fracture. While Ym1^+^ M2-like macrophages were also reduced in *Bgn* KO compared with WT mice, this was not significant until 2 days after injury (Figure 3B; Supplementary Figure S3). RNAseq analysis of the bones 3 days after fracture showed enhanced expression of inflammatory suppressor cytokines, such as IL-10 and SOCS3, in the *Bgn* KO (Figure 3C).

**FIGURE 4:**
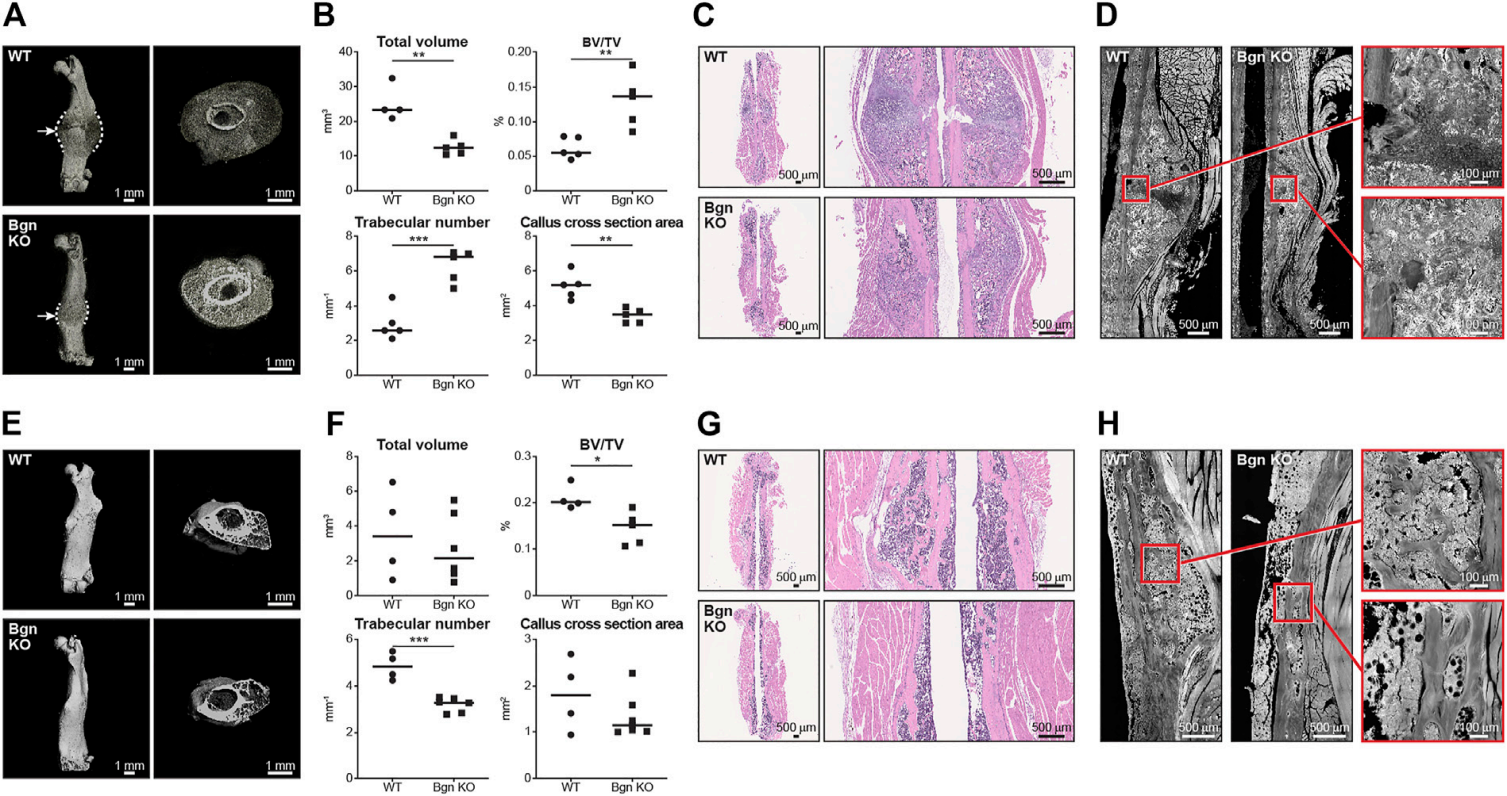
Lack of Bgn impairs bone healing after fracture. **(A)** 3D µCT reconstruction of femoral bone and cross section of the callus 2 weeks after fracture, representative images. Dashed lines mark the callus borders. Arrows marks the fracture site. **(B)** Quantitative µCT analysis of callus total volume, BV/TV, trabecular number and callus cross section area 2 weeks after fracture (n = 5 per group). **(C)** H&E staining of the callus area 2 weeks after fracture, representative images (*n* = 5 per group). **(D)** SHG representative images of healing bone 2 weeks after fracture (*n* = 5 per group). **(E)** 3D µCT reconstruction of femoral bone and cross section of the callus 8 weeks after fracture, representative images. **(F)** Quantitative µCT analysis of callus total volume, BV/TV, trabecular number and callus cross section area 8 weeks after fracture (*n* = 4/5 per group). **(G)** H&E staining of the callus area 8 weeks after fracture, representative images (*n* = 4/5 per group). **(H)** SHG representative images of the newly regenerated bone 8 weeks after fracture (*n* = 4/5 per group).

Since the role of the inflammation is to trigger the activation and proliferation of periosteal progenitors, we next measured the size of the periosteum 3 days after fracture and found that the periosteum of *Bgn* KO mice didn’t expand to the same extent as the WT after fracture (Figure 3D). The periosteum has a crucial role in bone regeneration and therefore we wanted to determine whether the expanding PDCs express Bgn. mRNA analysis and immunohistochemistry showed that in response to fracture, Bgn expression is upregulated in the bone, particularly in the enlarging periosteum (Figures 3E, F). Since the rapid periosteal expansion leads to callus formation around the fracture site, we measured the callus formation 1, 2 and 8 weeks after fracture. In the earlier time point of 1 week after fracture, there were no significant differences in callus total volume, BV/TV, trabecular number and callus cross section between *Bgn* KO and WT mice (Supplementary Figure S4). However, 2 weeks post fracture, when the full callus is formed, we found that fractured bones from *Bgn* KO mice formed a smaller callus compared with WT mice (Figures 4A–D). µCT analysis demonstrated lower total callus volume and callus cross section area in *Bgn* KO fractured bones, whereas BV/TV and the trabecular number were higher (Figure 4B). In addition, massive cartilage tissue was observed in the WT callus compared with that from *Bgn* KO, as shown by both H&E staining (Figure 4C) and SHG imaging (Figure 4D), suggesting an impaired cartilage phase in the *Bgn* KO mice at this time point compared with the WT. These results concur with cartilage specific aggrecan (ACAN) staining we previously published (Berendsen et al., 2014). The amount of TRAP staining in the callus was not significantly different between WT and *Bgn* KO bones 2 weeks after fracture (data not shown) indicating that the smaller callus in the KO bones isn’t due to higher bone resorption. By week 8, most of the callus formed in the *Bgn* KO fractured bones was resorbed and the bone had prematurely remodeled into mature bone (Figures 4E–H). µCT analysis 8 weeks post fracture showed there was higher BV/TV and trabecular number in the callus of the fractured WT bones compared with *Bgn* KO bones with no significant differences in the callus total volume and cross section area (Figure 4F). Furthermore, analysis of the newly formed bone by both H&E staining and SHG imaging (Figures 4G, H respectively) showed that WT bones developed more uniform and mature collagen fibril-like structures compared with the healing *Bgn* KO bone. Analyzing the SHG images of 8 weeks post fracture bones, revealed that both the collagen fiber length and width were higher in the regenerated *Bgn* KO bones, whereas no differences were found in collagen fiber number (Supplementary Figure S5A). When we analyzed WT and *Bgn* KO in un-fractured bones, there were no differences in the number, length and width of the collagen fibers (Supplementary Figure S5B), however the collagen fiber orientation wasn’t normal. A closer look at irregular regions of Bgn KO bones using second harmonic microscopy, showed significant changes in the structure, that may have an overall impact on bone function (Figures 2C, D). We take these observations to indicate that the small callus formed by the *Bgn* KO arises from an abnormal progression of the healing process that results in bone with inferior structure.

#### Bgn affects cartilage and bone differentiation in 3D culture system

Since PDCs have an important role in the healing process, we decided to study their activity using a 3D culture system in order to more closely mimic the native environment of the cells.

**FIGURE 5:**
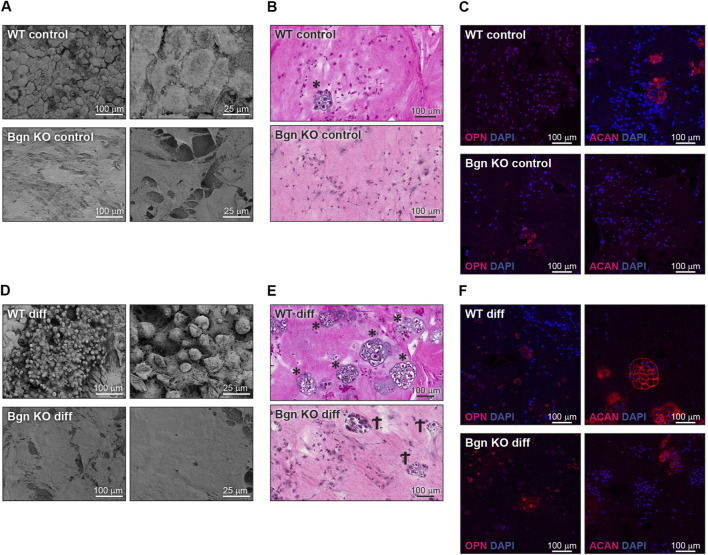
Bgn affects cartilage and bone differentiation in 3D culture system. **(A)** SEM images of the ECM secreted by undifferentiated (control) PDCs in the 3D structures, representative images (*n* = 5 per group). **(B)** H&E staining of the control 3D structures representative images (*n* = 5 per group). * marks chondrocytes. **(C)** Immunostaining of the control 3D structures stained for ACAN and OPN, representative images. See also Figure S5A-C (*n* = 5-8 per group). **(D)** SEM images of the ECM secreted by the differentiated PDCs in the 3D structure, representative images (*n* = 5 per group). **(E)** H&E staining of the differentiated 3D structures, representative images (*n* = 5 per group). * marks chondrocytes. Ϯ marks dead cells. **(F)** Immunostaining of the differentiated 3D structures stained for ACAN and OPN, representative images. See also Supplementary Figures S5A–C (*n* = 5-8 per group).

The 3D RAFT™ cell culture is based on rat tail collagen type I, where the cells and the collagen are mixed together to form a 100 µ thick hydrogel disc, in the size of a well in a 96 well plate. After isolation, PDCs from WT and *Bgn* KO mice were allowed to proliferate within the 3D culture system and either maintained with standard culture medium or induced towards osteogenic differentiation for 2 weeks. Analysis of the control (un-differentiated) 3D scaffolds with scanning electron microscopy (SEM) showed discrete differences between the ECM secreted by WT and *Bgn* KO PDCs (Figure 5A). H&E staining of the WT control 3D cultures surprisingly showed some chondrocyte clusters, a finding which was confirmed by aggrecan (ACAN) immunostaining. Neither chondrocytes nor aggrecan staining was detected in *Bgn* KO control 3D structures (Figures 5B, C; Supplementary Figure S6A). Upon osteogenic differentiation in the 3D culture system, WT PDCs seemed to undergo endochondral ossification by secreting ACAN and forming cartilage structures that were incorporated into the matrix (Figures 5D–F; Supplementary Figure S6A). At the same time, evidence of dead chondrocytes was found in the differentiated *Bgn* KO PDCs as shown by H&E staining and low level of ACAN staining (Figures 5E, F; Supplementary Figure S6A). The total number of cells (quantified by DAPI staining) wasn’t significantly different between *Bgn* KO and WT 3D constructs (Supplementary Figure S6C).

**FIGURE 6:**
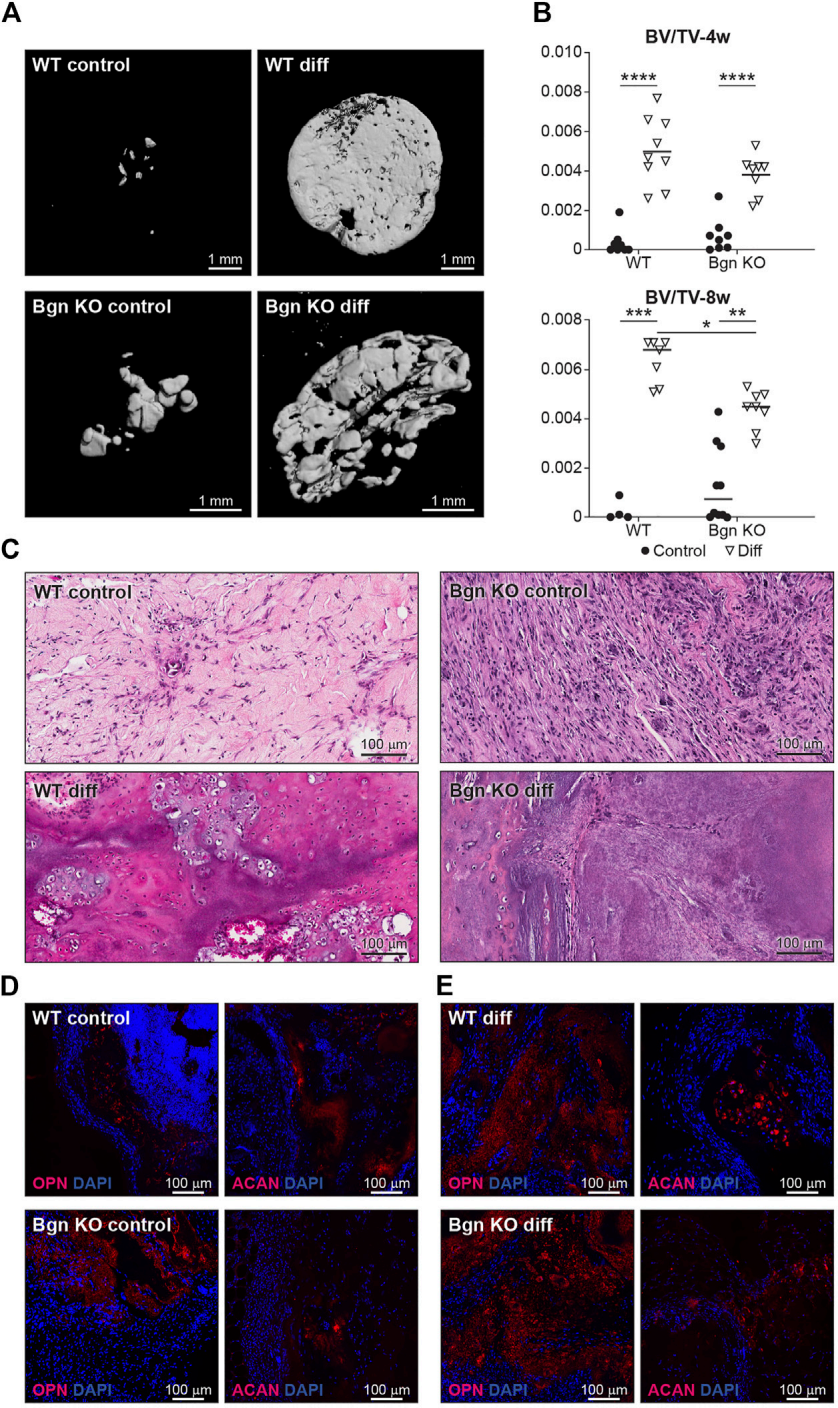
Lack of *Bgn* in a 3D culture system result in compromised bone formation. **(A)** 3D µCT reconstruction of the implants 8 weeks after subcutaneous implantation, representative images. **(B)** Quantitative µCT analysis of BV/TV of the 3D structures 4 and 8 weeks after subcutaneous implantation. **(C)** H&E staining of the 3D structures 8 weeks after subcutaneous implantation, representative images. **(D)** Immunostaining of the control 3D structures stain for ACAN and OPN 8 weeks after subcutaneous implantation, representative images. See also Supplementary Figures S5D–F (*n* = 4-9 per group). **(E)** Immunostaining of the differentiated 3D structures stained for ACAN and OPN 8 weeks after subcutaneous implantation, representative images. See also Supplementary Figures S5D–F (*n* = 4-9 per group).

When the 3D structures were subcutaneously implanted into syngeneic mice, we found that the differentiated PDCs (both WT and KO) created mineralized tissues 4 and 8 weeks post implantation (Figures 6A, B). To understand the composition of the mineralized 3D structures 8 weeks post transplantation, histological analysis was performed. Differentiated WT implants displayed an endochondral ossification pattern with chondrocytes surrounded by bone and blood vessels, which wasn’t found in the structures formed by differentiated *Bgn* KO PDCs (Figure 6C). Since the H&E staining of the *Bgn* KO implants was difficult to interpret, immunofluorescence staining for ACAN and OPN was performed. The WT control implants showed again higher expression of ACAN compared with the *Bgn* KO control implants (Figure 6D; Supplementary Figure S6D). As expected, after 8 weeks of implantation, differentiated implants from both genotypes showed some staining for ACAN and intense staining for OPN, however, *Bgn* KO implants demonstrated more robust staining of OPN compared with WT (Figure 6E; Supplementary Figure S6E). No differences were observed in cell number between the samples as quantified by DAPI staining (Supplementary Figure S6F).

The low level of cartilage differentiation coupled with a high level of OPN expression suggest that in the absence of *Bgn*, the cartilage phase preceding bone is compromised, leading to more direct bone differentiation.

## Discussion

Bone tissue has the unique ability to heal and regenerate throughout life. This process, which is similar to embryonic endochondral bone development, relies on a complex sequence of physiological events leading to the formation of new bone at the fracture site. The inflammatory response is necessary in the early stages of bone healing to trigger and activate the proliferation of skeletal progenitor cells within the periosteal layer. During healing, the differentiation of progenitor cells into chondrocytes and osteoblasts gives rise to a callus around the fracture site. As the chondrocytes undergo hypertrophy, blood vessels and osteoprogenitor cells are attracted from nearby periosteal regions. The cartilage template is degraded and replaced by woven bone which ultimately remodels to the mature bone (Marsell and Einhorn, 2011; Roberts et al., 2015; Salhotra et al., 2020). In this study, we demonstrate a role for *Bgn* in early stages of bone development, as well as in bone repair.

The *Bgn* gene is located on the X chromosome in mice and humans and is highly expressed during development (Bianco et al., 1990; Fisher et al., 1991; Wilda et al., 2000). Recently, loss-of-function mutations in the human *Bgn* gene were found in Meester-Loeys syndrome (MRLS) (Meester et al., 2017). Clinically, MRLS patients present with early-onset aortic aneurysm and dissection. The males carrying the deletion also have skeletal dysplasia, which is characterized by relatively short stature, phalangeal dysplasia, brachydactyly, hip dislocation and dysplastic epiphyses of the long bones. Like MRLS patients, *Bgn* KO mice also have skeletal abnormalities. *Bgn*-deficient mice acquire early onset osteoporosis-like phenotype with significant decreases to their trabecular bone volume and mineral apposition rate (Xu et al., 1998; Chen et al., 2002), as well as structural abnormalities of collagen fibrils in bone, dermis, and tendon (Corsi et al., 2002). While these studies point to the importance of Bgn in bone, its exact role in skeletal healing and early bone formation was unclear.

Bone is a heterogeneous organ, composed of a variety of cell types. Single-cell sequencing is an advanced technique that can be used to understand the cellular basis of skeletal development (Greenblatt et al., 2019). In order to detect the onset of possible changes in the cell landscape in *Bgn* KO mice during embryonic development, single cell RNAseq analysis of E16.5 WT and *Bgn* KO embryo hindlimbs was performed for the first time. Seurat’s unbiased cluster detection algorithm defined 38 cell populations within the long bone endocortical samples of both WT and *Bgn* KO. Our analysis revealed 4 osteoblast clusters (1–4). Compared to clusters 1 and 2, clusters 3 and 4 expressed elevated levels of osteogenic genes, which were identified by GO analysis, and were sequentially located after cluster 1 on the UMAP pseudo-time trajectory branch. This data suggests that cells in cluster 4 are more committed and mature osteoblasts compared with those found in clusters 1-3. We show that the majority of cells that populate the mature cluster 4 originate from WT samples. We hypothesize that the lower levels of mature osteoblast cells found in *Bgn* KO mice, as early as 16.5 days of embryonic development, together with a reduced number of cells in the chondrocyte clusters, leads to the abnormalities seen in the adult *Bgn* KO bones. Previous *in vitro* studies showed an increased number of osteoclasts in adult *Bgn* KO mice which was presumed to be due to defects in the proliferation and differentiation of osteoblasts and their precursors (Bi et al., 2006). Our studies established, at the single-cell level, the concept that *Bgn* is needed for embryonic bone-cell maturation, and that the absence of Bgn could, later in life, result in defective osteogenesis. Further experiments to validate our findings at the protein level will be needed to confirm this theory. Our lab has previously found that Bgn’s core protein enhances canonical Wnt signaling (Berendsen et al., 2011). Moreover, using other skeletal progenitors, we showed that *Bgn* can regulate TGF-β activity (Chen et al., 2002; Bi et al., 2007; Embree et al., 2010). Both pathways could be important mechanisms to modulate *Bgn* skeletal cell differentiation and cell fate.

In mature bones, we show here the importance of *Bgn* to bone integrity, where *Bgn* KO bones have reduced cortical thickness and trabecular number, and an increase in trabecular spacing and bone mineral content (BMC), leading to a more fragile bone that is biomechanically compromised. Additionally. We show that *Bgn* is crucial for normal bone repair in response to injury. Shortly after bone fracture, chemokines and inflammatory cytokines are secreted to recruit inflammatory cells and promote angiogenesis. It was previously shown that *Bgn* can serve as a pro-inflammatory factor in a sepsis model and the mechanistic basis involves TLR 2 and 4 (Schaefer et al., 2005). In the present study, we demonstrate that *Bgn* plays a regulatory role in the immune response during the first phase of fracture healing. Mice lacking *Bgn* had a decreased inflammatory response demonstrated by decreased secretion of inflammatory cytokines, MCP-1 and IL-6, and reduced macrophage infiltration around the fracture site. MCP-1 (also known as CCL2), which has been shown to be expressed in the periosteum around the fracture site during fracture healing (Edderkaoui, 2017), is one of the earliest, highly expressed chemokines in response to fracture and is involved in regulating angiogenesis and macrophage infiltration. IL-1 and IL-6 are believed to be critical cytokines for fracture healing. IL-1, produced by macrophages during the acute phase of inflammation, induces the production of IL-6 in osteoblasts. It also stimulates the formation of the initial cartilaginous callus and promotes angiogenesis around the fracture site. IL-6, also produced during the acute inflammatory phase, stimulates angiogenesis and vascular endothelial growth factor (VEGF) production (Kon et al., 2001; Mountziaris and Mikos, 2008; Marsell and Einhorn, 2011). Fracture healing requires a blood supply and therefore revascularization is essential for successful bone repair. We previously showed that compared with WT controls, *Bgn*-deficient mice have a significant decrease in VEGF gene expression and concomitant smaller vessel size and volume around the fracture site (Berendsen et al., 2014; Myren et al., 2016). The reduced inflammatory response and angiogenesis in the *Bgn* KO mice may be the cause for the observed reduction in periosteal expansion around the fracture site, resulting in the formation of a smaller callus 14 days post fracture. We found that the smaller callus in *Bgn* KO mice has substantially fewer chondrocytes compared with WT, which is in agreement with previous studies showing less aggrecan at the callus site of *Bgn* KO mice compared with WT (Berendsen et al., 2014). µCT analysis 14 days after fracture surprisingly demonstrated higher BV/TV and trabecular number in the *Bgn* KO callus compared with WT, which may indicate that the defective cartilage phase in *Bgn* KO mice forced the healing process to occur through direct bone development. 8 weeks post fracture, the *Bgn* KO callus was almost completely resolved, excluding the possibility the healing process in the KO mice is delayed. We believe that the larger the callus is, the more time it takes for it to resolve and heal. Since *Bgn* KO have a smaller callus, it takes less time for it to fully heal, which does not necessarily mean the process itself is accelerated.

The periosteum is a major source of the heterogenous array of skeletal progenitor cells, important not only in bone development but also during fracture healing. Several studies demonstrated that removal of the periosteum dramatically impairs bone repair (Ozaki et al., 2000; Colnot, 2009). Ozaki et al. showed a delay in cartilage formation after fracture when the periosteum was removed, suggesting that the periosteum and its PDCs are important for mediating chondrogenesis during the endochondral ossification phase in bone repair (Ozaki et al., 2000). In this study, we found that *Bgn* is highly expressed in the expanding periosteum after fracture. *Bgn*-deficient periosteum didn’t expand to the same extent as the WT, leading to smaller callus formation. Our results clearly demonstrate the importance of *Bgn* in the early phase of inflammation and during the subsequent periosteal expansion that is required for proper callus formation and proper healing during bone regeneration. These findings suggest that Bgn may influence bone repair by: 1) its pro-inflammatory role in the healing process and/or 2) by directly affecting the PDCs themselves.

Currently, like progenitor cells derived from the bone marrow (BMSCs), it has been challenging to identify markers that are specific for PDCs. In recent years, several markers have been proposed to identify PDCs, including Cathepsin K, periostin and alpha smooth muscle actin (αSMA), and it is likely these markers define subpopulations of the cells within the periosteal layer (Marecic et al., 2015; Debnath et al., 2018; Deveza et al., 2018; Duchamp de Lageneste et al., 2018; Ortinau et al., 2019; Matthews et al., 2021). To understand the role of Bgn on PDCs in their natural heterogenous state, we harvested the periosteum and used the entire cell population. A 3D culture system was employed to further mimic the native environment of the cells. We allowed the cells to proliferate on the 3D structures before subjecting them to osteogenic differentiation. After 2 weeks of differentiation, WT PDC cultures formed ACAN expressing chondrocytes, the cells that provide an essential template for bone repair and bone development. In the 3D cultures containing *Bgn* KO cells, the chondrogenic phase was bypassed and instead the cultures showed expedited bone development, as judged by the high level of OPN, a marker of early osteogenic differentiation. We further show, using a subcutaneous transplantation approach, that PDCs from WT and *Bgn* KO differentiate differently during endochondral ossification *in vivo*. Histological analysis of the calcified structures showed that compared with WT structures, which developed osteoblasts, chondrocytes and encouraged vascularization necessary for bone development, implants containing *Bgn* KO PDCs differentiated more directly into bone, expressing high levels of OPN. Although OPN is believed to be important for homeostasis of osteoclasts and osteoblasts, elevated levels of OPN are also functionally implicated in bone-related diseases, such as osteoporosis, rheumatoid arthritis, and osteosarcoma (Si et al., 2020). The high level of OPN expressed in the *Bgn* KO PDC cultures during osteogenic differentiation may be harmful to the structural integrity and strength of bone, which could be a potential basis for the early onset osteoporosis phenotype found in this genetic model (Xu et al., 1998).

In conclusion, *Bgn* deletion impairs endochondral bone formation and regeneration. The defective periosteal cells may be the key to the subsequent abnormalities we observed in the healing bone. More investigation will be required in the future to understand how *Bgn* and other ECM proteins influence skeletal stem cell populations during bone development and the periosteal progenitors during regeneration. Better understanding of these processes and their key elements will help in developing better strategies to treat skeletal defects and bone disease.

## Data Availability

The datasets presented in this study can be found in online repositories. The names of the repository/repositories and accession number(s) can be found below: https://www.ncbi.nlm.nih.gov/search/all/?term=GSE192542.
