# Supplementary figures and images for: Biglycan regulates bone development and regeneration
Source: Front Physiol. 2023 Feb 16;14:1119368. doi: 10.3389/fphys.2023.1119368 (PMC9979216; doi:10.3389/fphys.2023.1119368)

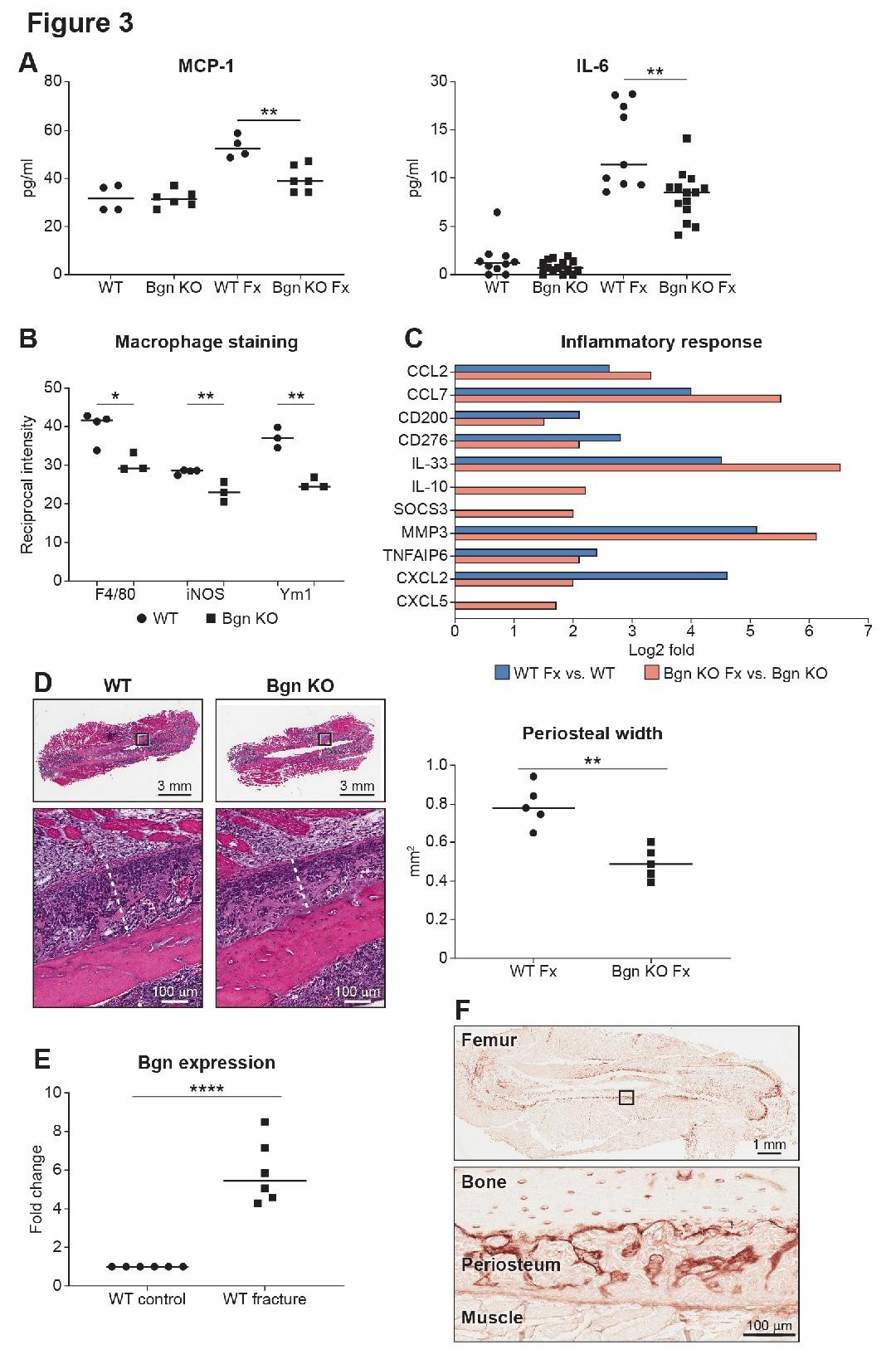

Supplement: Supplementary file 1 [file Image3.JPEG]

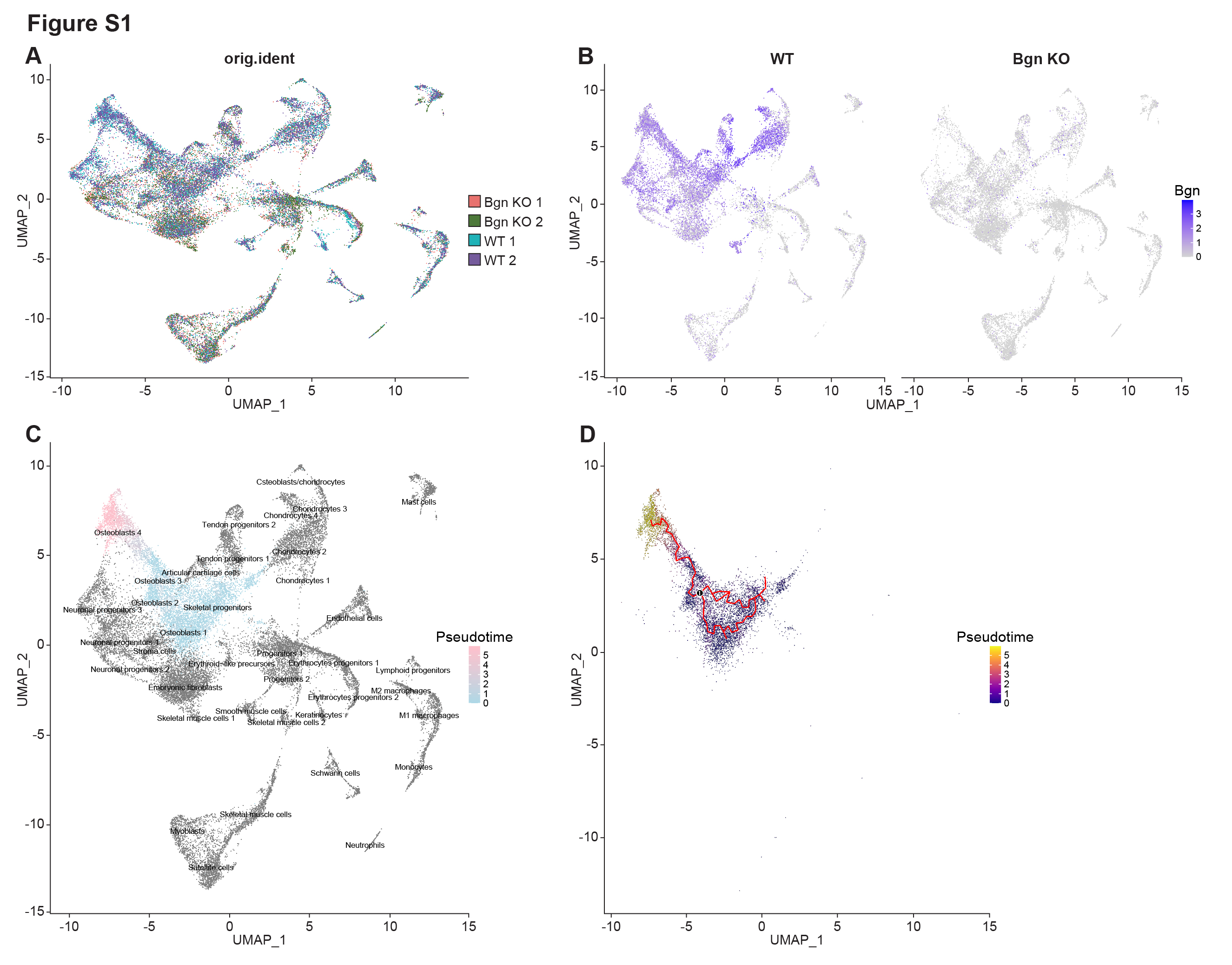

Supplement: Supplementary file 2 [file Image1.JPEG]

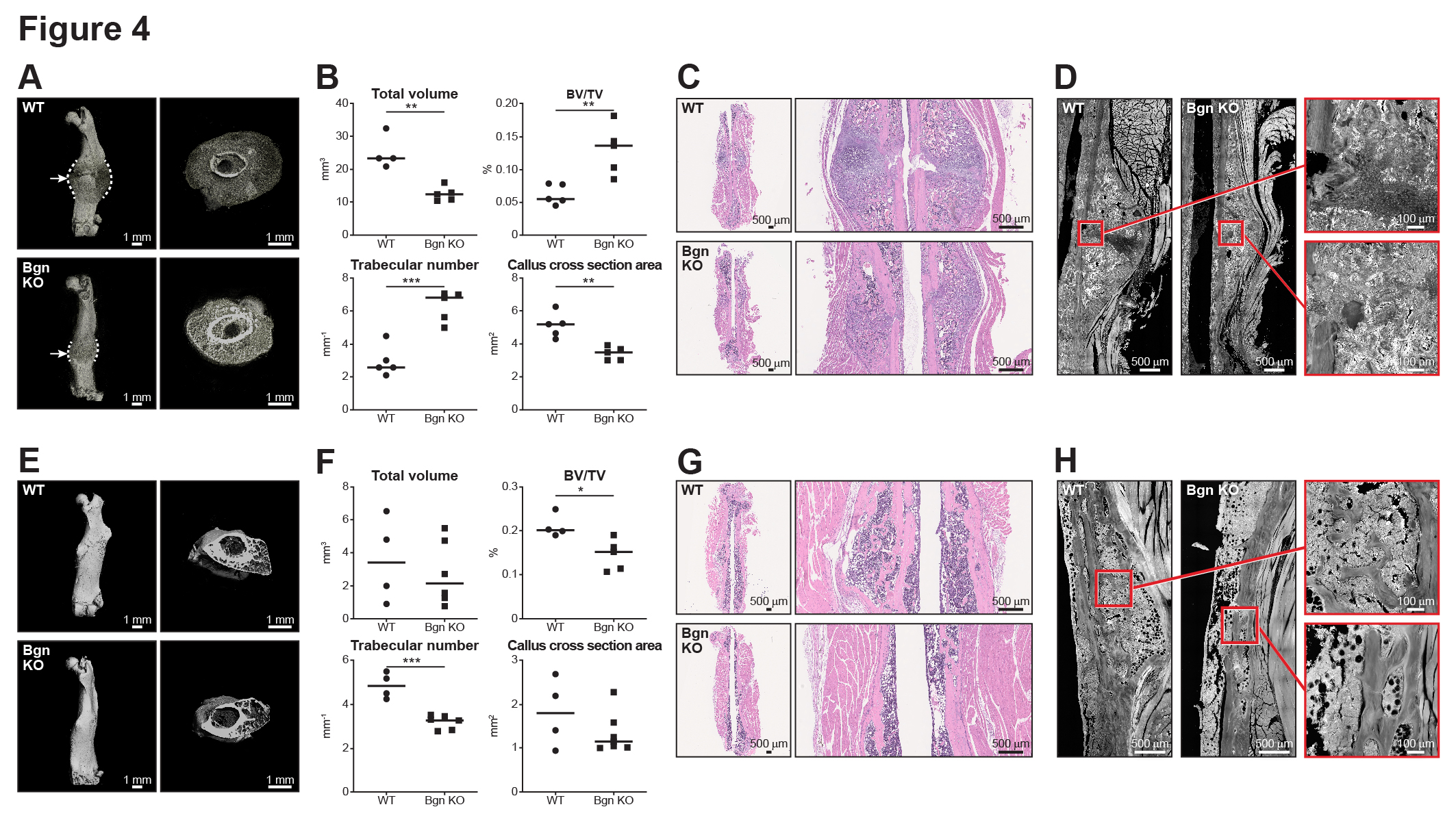

Supplement: Supplementary file 3 [file Image4.JPEG]

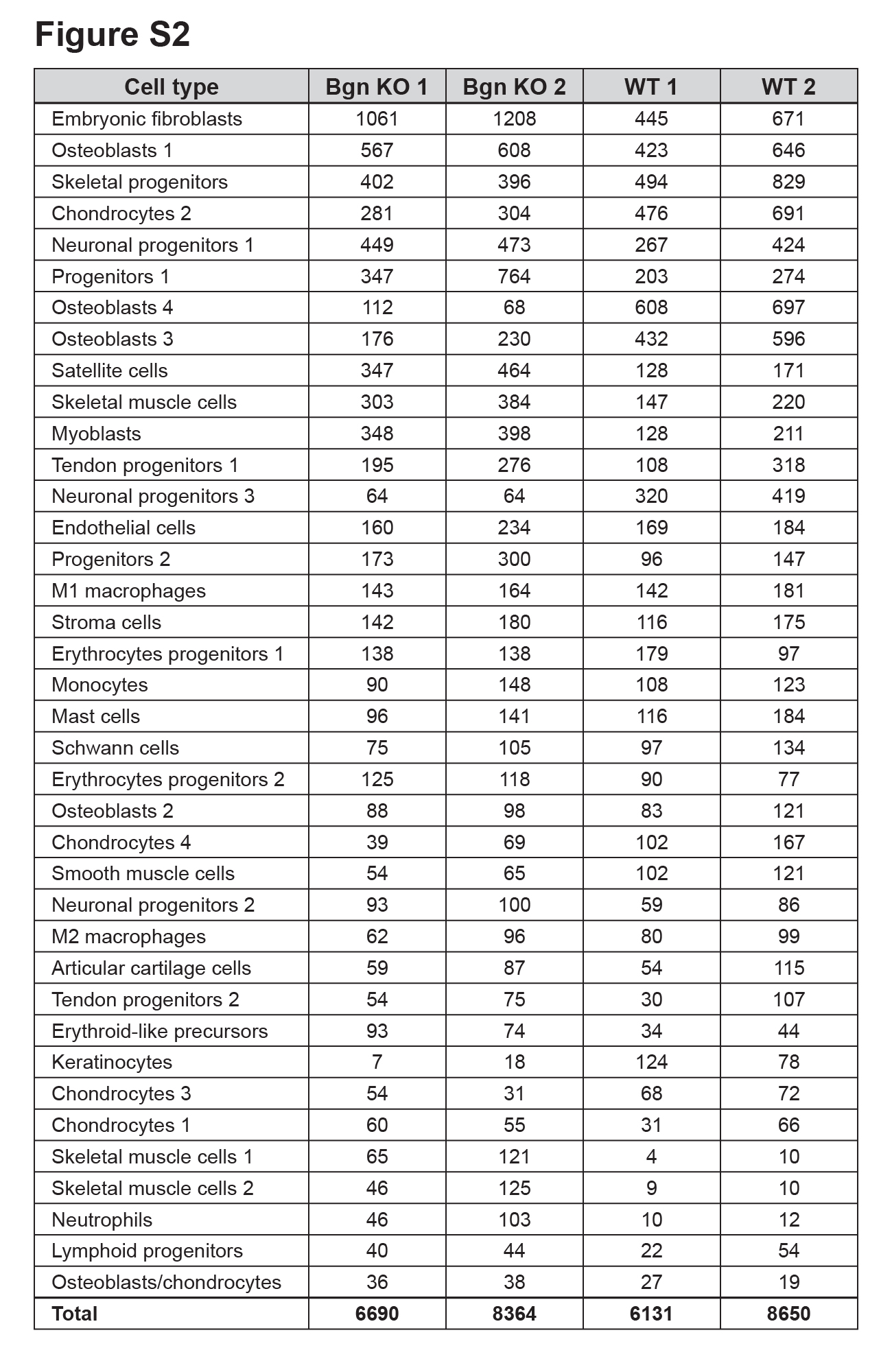

Supplement: Supplementary file 4 [file Image2.JPEG]

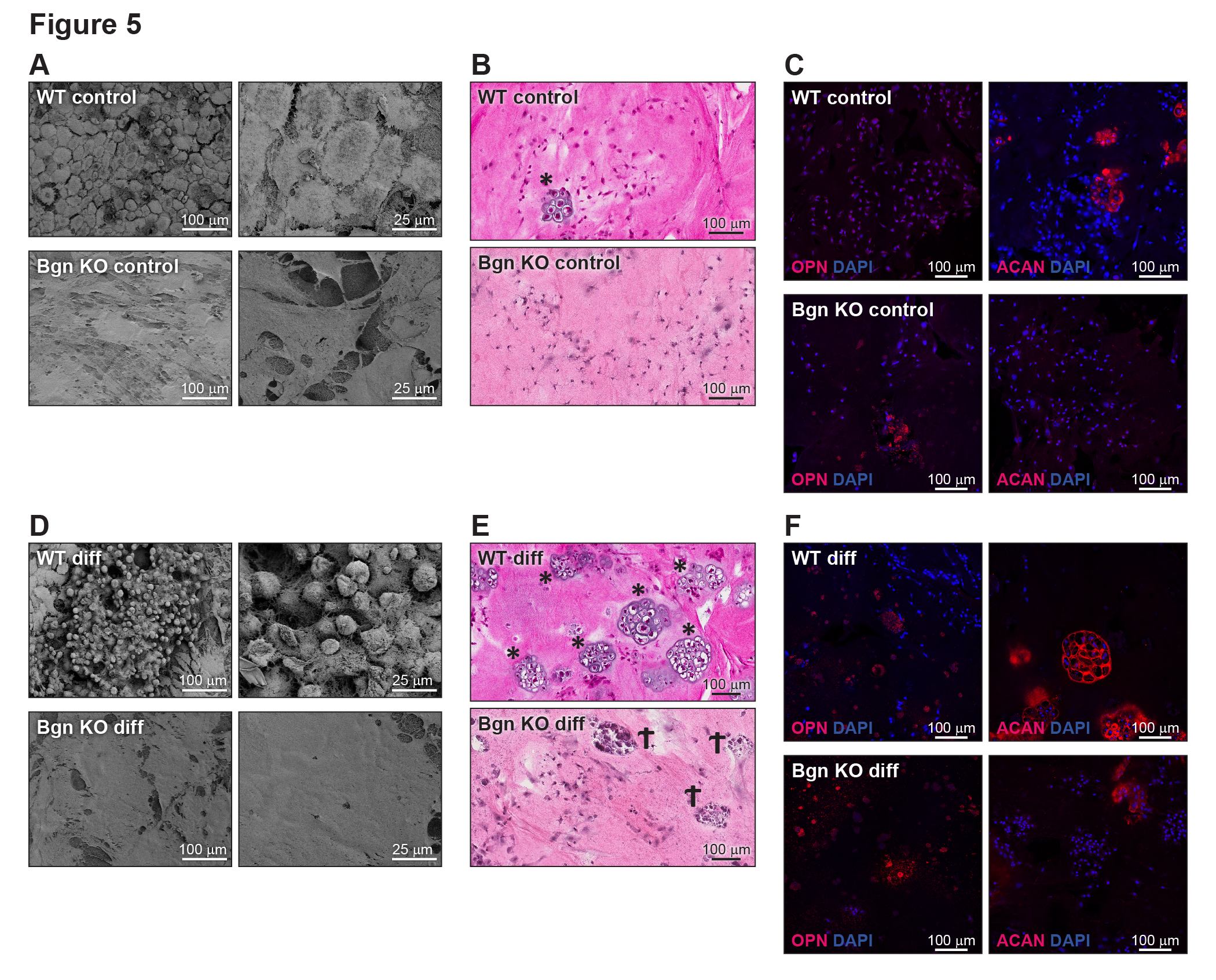

Supplement: Supplementary file 5 [file Image5.JPEG]

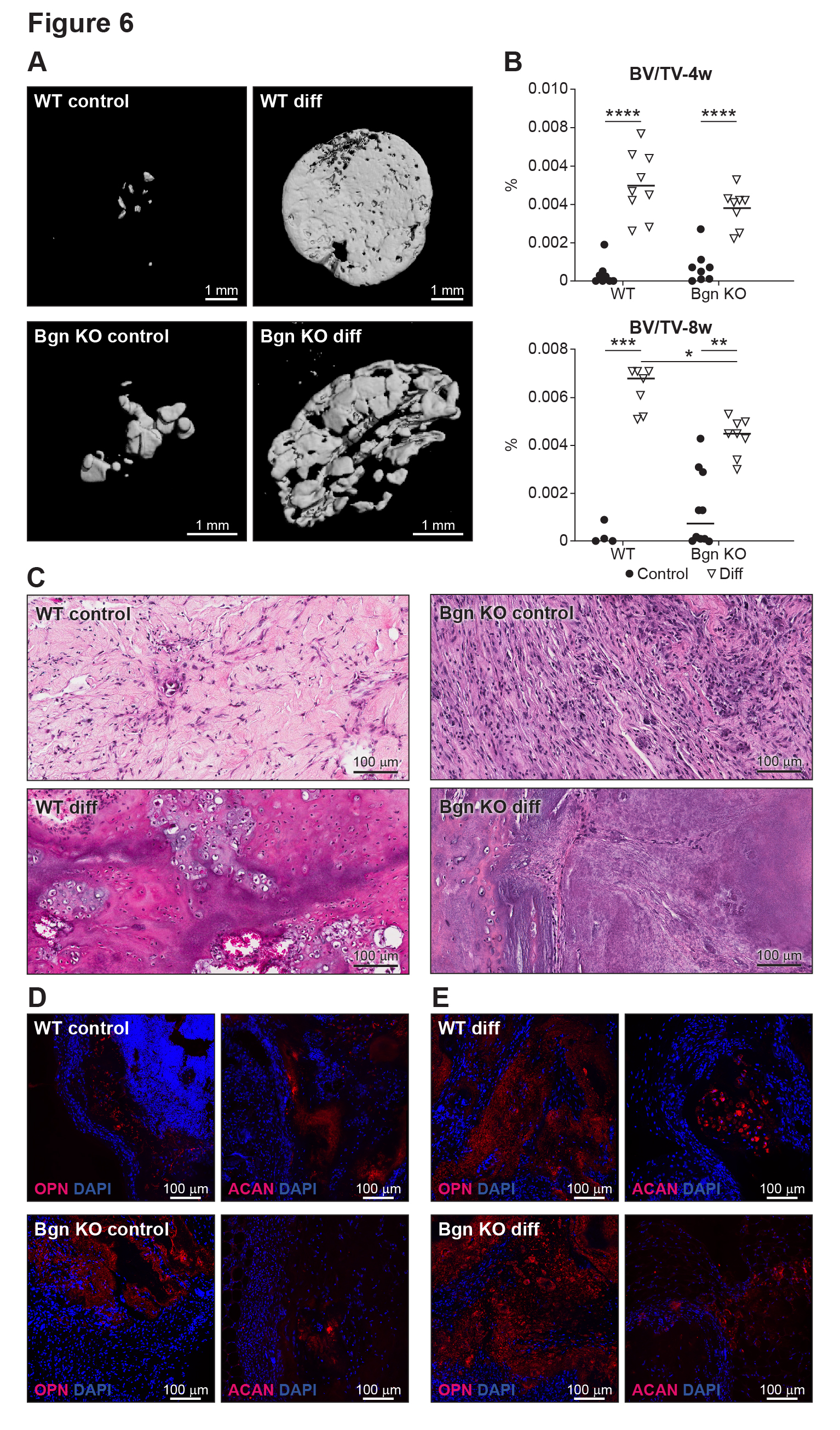

Supplement: Supplementary file 8 [file Image6.JPEG]
