# Supplementary material for: Biglycan regulates bone development and regeneration
Source: Front Physiol. 2023 Feb 16;14:1119368. doi: 10.3389/fphys.2023.1119368 (PMC9979216; doi:10.3389/fphys.2023.1119368)
Supplement: Supplementary file 6 [file DataSheet1.PDF]

Supplementary Material

Biglycan Regulates Bone Development and Regeneration

Reut Shainer[1], Vardit Kram[1], Tina M. Kilts[1], Li Li[1], Andrew D. Doyle[2], Inbal Shainer[3], Daniel Martin[4], Genomics and Computational Biology Core[4], Carl G. Simon Jr.[5], Jinyang Zeng-Brouwers[6], Liliana Schaefer[6], Marian F. Young[1]*.

[1]Molecular Biology of Bones and Teeth Section, National Institute of Dental and Craniofacial Research, National Institutes of Health, Bethesda, MD 20892, USA.

[2]NIDCR Imaging Core, National Institute of Dental and Craniofacial Research, National Institutes of Health, Bethesda, MD 20892, USA.

[3]Max Planck Institute for Biological Intelligence, Department Genes-Circuits-Behavior, Martinsried, 82152, Germany.

[4]NIDCD/NIDCR Genomics and Computational Biology Core, National Institutes of Health, Bethesda, MD 20892, USA.

[5]Biosystems and Biomaterials Division, National Institute of Standards and Technology, Gaithersburg, MD 20899, USA.

[6]Pharmazentrum Frankfurt, Institut für Allgemeine Pharmakologie und Toxikologie, Klinikum der Goethe-Universität Frankfurt am Main, Frankfurt am Main 60590, Germany.

*Corresponding author: myoung@dir.nidcr.nih.gov

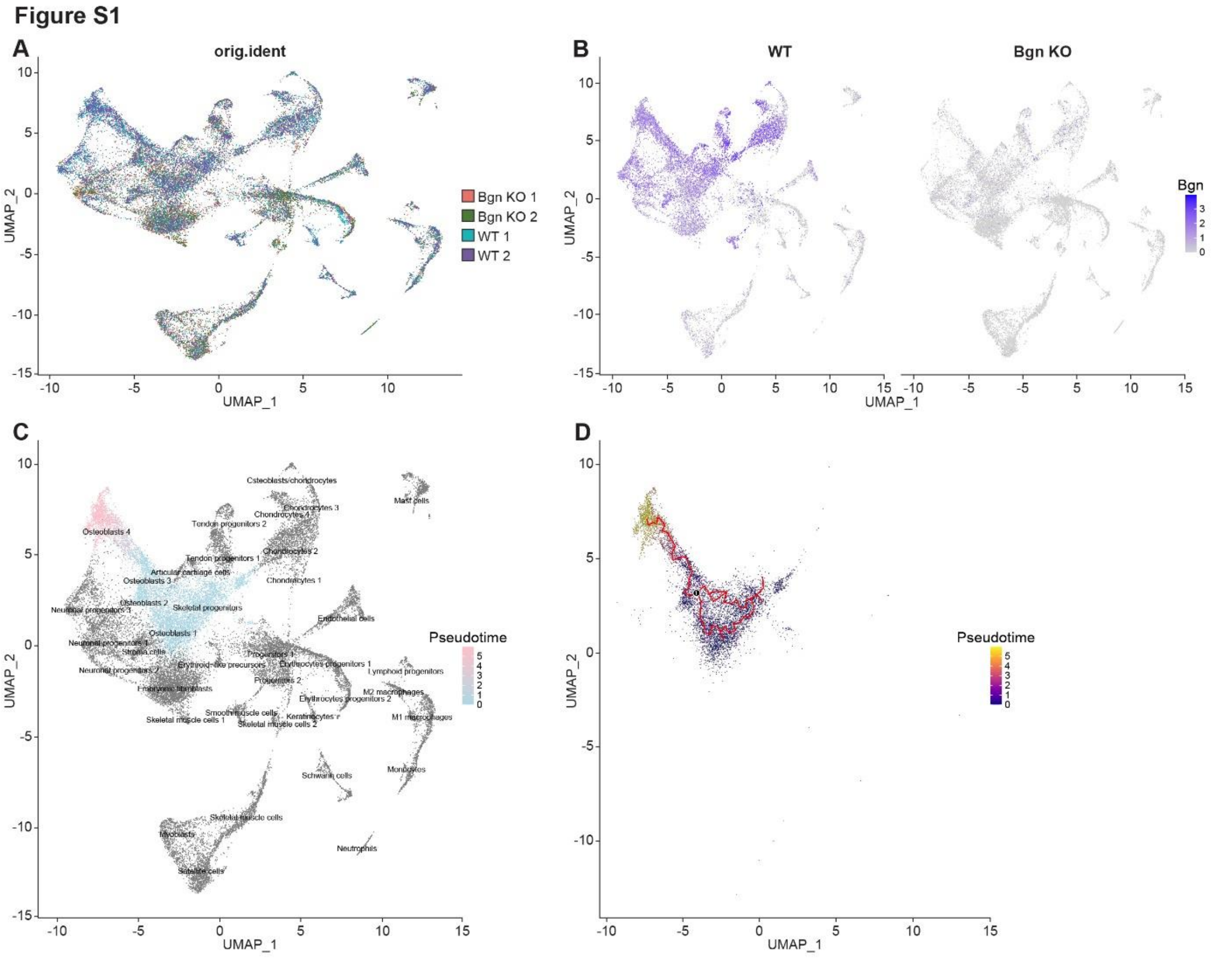

Figure S1. Single-cell sequencing of mouse embryonic bones shows Bgn is important for osteoblast differentiation.

(Related to Figure 1)

(A) UMAP of the cells according to their genotype origin and biological replica.

(B) UMAP visualization of Bgn gene expression split according to the genotype origin.

(C) The pseudotime trajectory analysis of the osteoblast clusters within the entire UMAP dataset.

(D) Pseudotime trajectory analysis of the osteoblast clusters. The skeletal progenitors were defined as the root. A single branching point (labeled as 1) was detected, leading the cell fate towards osteoblasts 3 and osteoblasts 4. Distance from the root point (skeletal progenitors) represent the pseudotime, while branching points represent cell fate decisions.

Figure S2

Cell type	Bgn KO 1	Bgn KO 2	WT 1	WT 2
Embryonic fibroblasts	1061	1208	445	671
Osteoblasts 1	567	608	423	646
Skeletal progenitors	402	396	494	829
Chondrocytes 2	281	304	476	691
Neuronal progenitors 1	449	473	267	424
Progenitors 1	347	764	203	274
Osteoblasts 4	112	68	608	697
Osteoblasts 3	176	230	432	596
Satellite cells	347	464	128	171
Skeletal muscle cells	303	384	147	220
Myoblasts	348	398	128	211
Tendon progenitors 1	195	276	108	318
Neuronal progenitors 3	64	64	320	419
Endothelial cells	160	234	169	184
Progenitors 2	173	300	96	147
M1 macrophages	143	164	142	181
Stroma cells	142	180	116	175
Erythrocytes progenitors 1	138	138	179	97
Monocytes	90	148	108	123
Mast cells	96	141	116	184
Schwann cells	75	105	97	134
Erythrocytes progenitors 2	125	118	90	77
Osteoblasts 2	88	98	83	121
Chondrocytes 4	39	69	102	167
Smooth muscle cells	54	65	102	121
Neuronal progenitors 2	93	100	59	86
M2 macrophages	62	96	80	99
Articular cartilage cells	59	87	54	115
Tendon progenitors 2	54	75	30	107
Erythroid-like precursors	93	74	34	44
Keratinocytes	7	18	124	78
Chondrocytes 3	54	31	68	72
Chondrocytes 1	60	55	31	66
Skeletal muscle cells 1	65	121	4	10
Skeletal muscle cells 2	46	125	9	10
Neutrophils	46	103	10	12
Lymphoid progenitors	40	44	22	54
Osteoblasts/chondrocytes	36	38	27	19
**Total**	**6690**	**8364**	**6131**	**8650**

Figure S2. Bgn is important for osteoblast differentiation during embryonic bone development.

(Related to Figure 1).

The number of cells in each of the clusters based on their genotype.

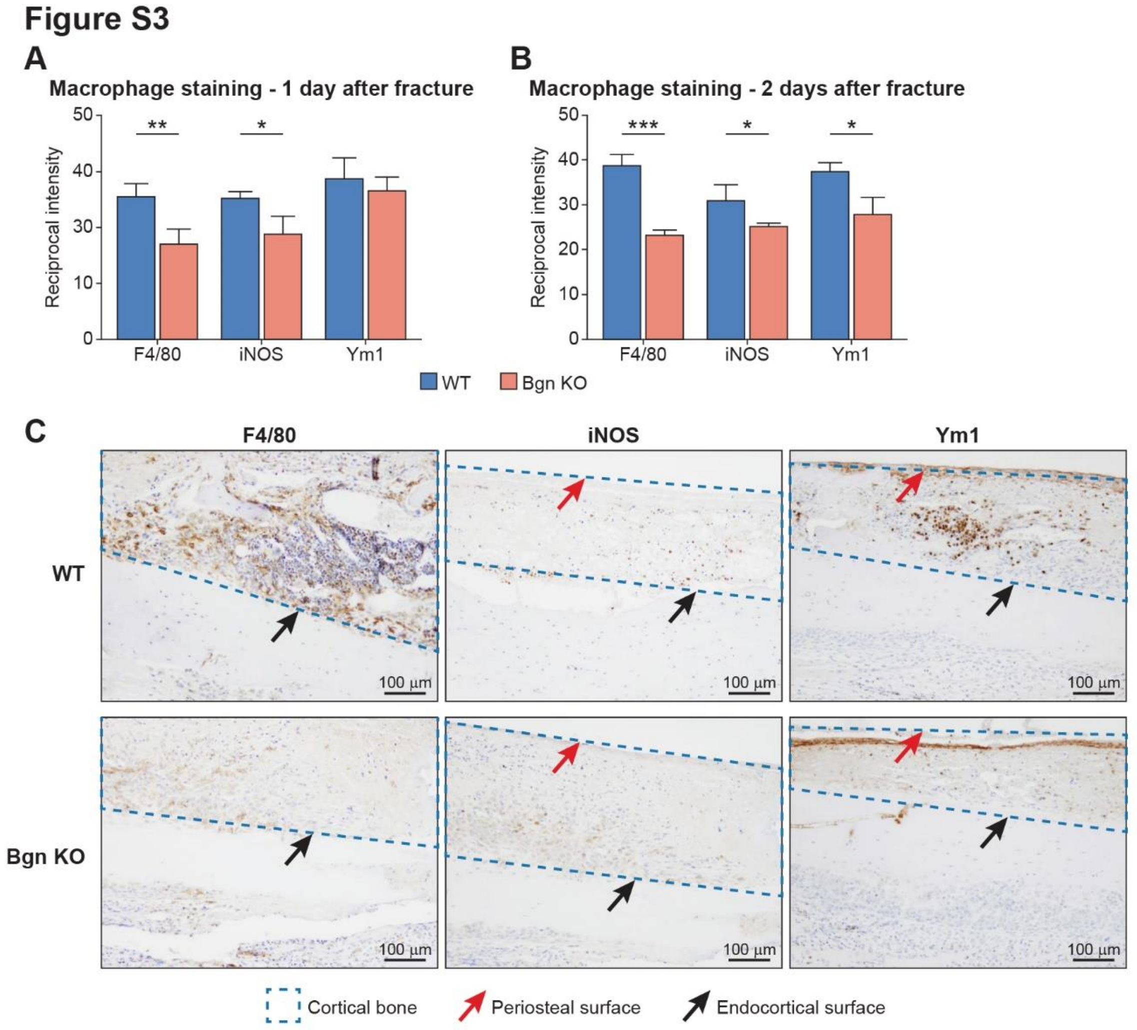

Figure S3. Bgn is needed for the inflammatory response after fracture.

(Related to Figure 3)

(A) Quantification of immunostaining for F4/80, iNOS and Ym1 around the fracture site 24 h after fracture. (n = 3 per group).

(B) Quantification of immunostaining for F4/80, iNOS and Ym1 around the fracture site 48 h after fracture. (n = 3 per group).

(C) Representative images of the immunostaining for F4/80, iNOS and Ym1 around the fracture site 72 h after fracture. (n = 3 per group).

Figure S4

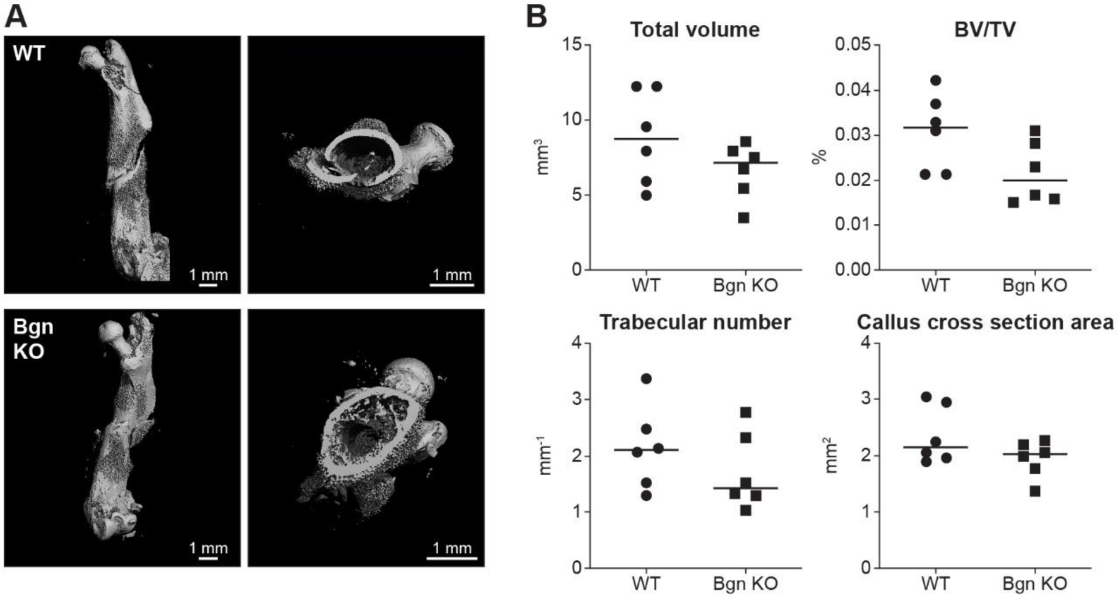

Figure S4. μCT analysis 7 days post fracture shows no significant differences in bone healing.

(Related to Figure 4)

(A) 3D μCT reconstruction of femoral bone and cross section of the callus 1 week after fracture, representative images.

(B) Quantitative μCT analysis of callus total volume, BV/TV, trabecular number, and callus cross section area 1week after fracture. (n = 6 per group).

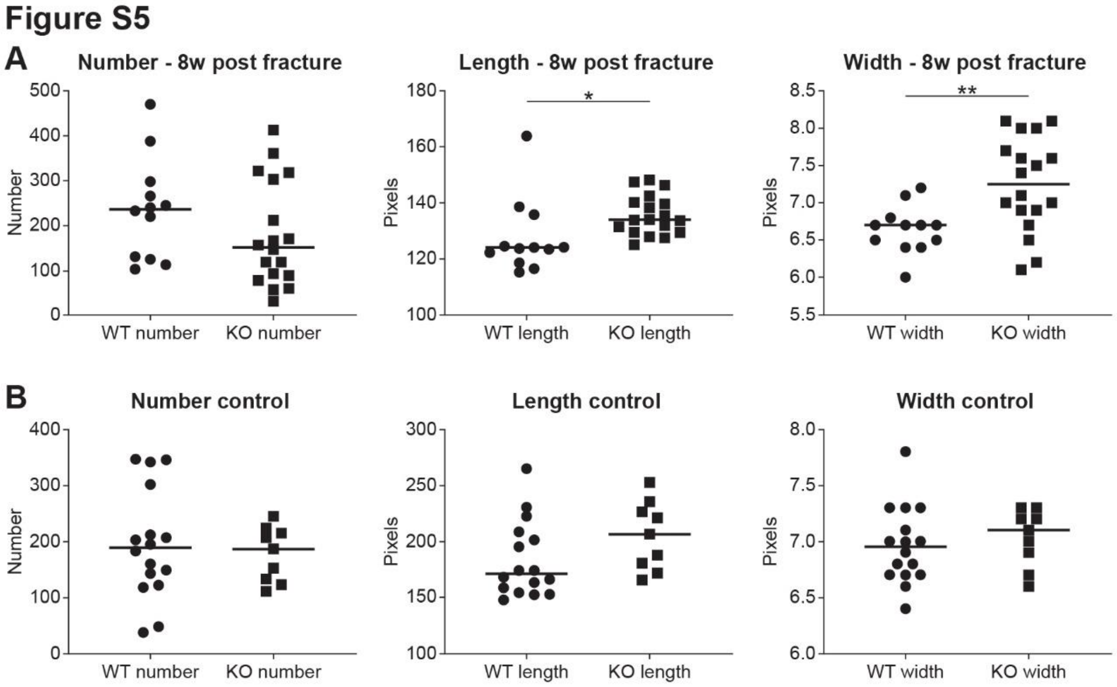

Figure S5. Lack of Bgn impairs bone healing after fracture

(Related to Figure 4)

(A) Quantitative SHG analysis of Type I collagen images of non-fractured bones.

(B) Quantitative SHG analysis of Type I collagen images of the newly regenerated bone 8 weeks after fracture.

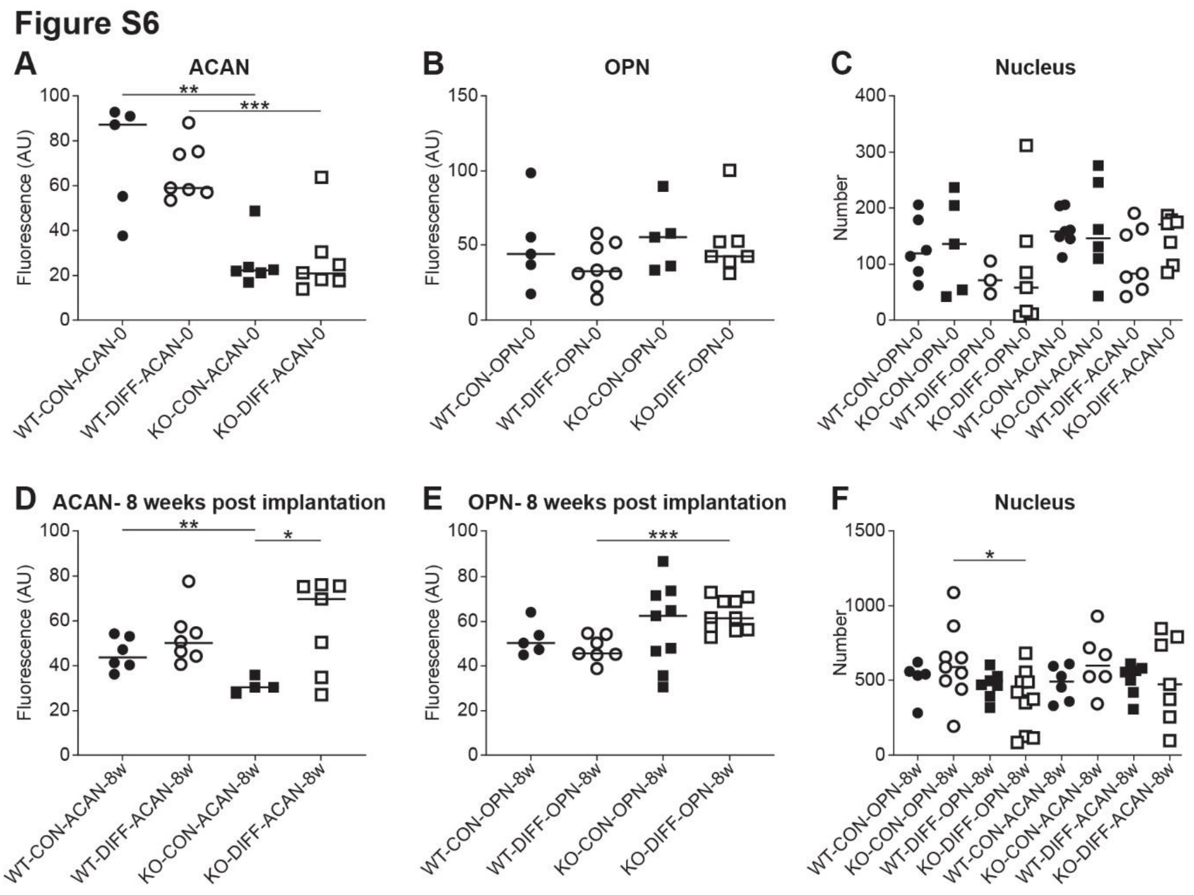

Figure S6. Bgn affects cartilage and bone differentiation in a 3D culture system.

(Related to Figure 5 and Figure 6)

(A) Quantification of the ACAN immunostaining in the 3D structures on day of implantation.

(B) Quantification of the OPN immunostaining in the 3D structures on day of implantation.

(C) Cell number (based on DAPI staining) in the 3D structures ACAN and OPN on day of implantation.

(D) Quantification of the ACAN immunostaining in the 3D structures 8 weeks after subcutaneous implantation.

(E) Quantification of the OPN immunostaining in the 3D structures 8 weeks after subcutaneous implantation.

(F) Cell number (based on DAPI staining) in the 3D structures stained for ACAN and OPN 8 weeks after subcutaneous implantation.
